# Deep sequencing of the viral *phoH* gene reveals temporal variation, depth-specific composition, and persistent dominance of the same viral *phoH* genes in the Sargasso Sea

**DOI:** 10.7717/peerj.997

**Published:** 2015-06-16

**Authors:** Dawn B. Goldsmith, Rachel J. Parsons, Damitu Beyene, Peter Salamon, Mya Breitbart

**Affiliations:** 1 College of Marine Science, University of South Florida, St. Petersburg, FL, USA; 2 Bermuda Institute of Ocean Sciences, St. George’s, Bermuda; 3 Department of Mathematics and Statistics, San Diego State University, San Diego, CA, USA

**Keywords:** *phoH*, Sargasso Sea, Viral diversity, Bermuda Atlantic Time-series Study

## Abstract

Deep sequencing of the viral *phoH* gene, a host-derived auxiliary metabolic gene, was used to track viral diversity throughout the water column at the Bermuda Atlantic Time-series Study (BATS) site in the summer (September) and winter (March) of three years. Viral *phoH* sequences reveal differences in the viral communities throughout a depth profile and between seasons in the same year. Variation was also detected between the same seasons in subsequent years, though these differences were not as great as the summer/winter distinctions. Over 3,600 *phoH* operational taxonomic units (OTUs; 97% sequence identity) were identified. Despite high richness, most *phoH* sequences belong to a few large, common OTUs whereas the majority of the OTUs are small and rare. While many OTUs make sporadic appearances at just a few times or depths, a small number of OTUs dominate the community throughout the seasons, depths, and years.

## Introduction

Viruses are the most abundant biological entities on the planet (Breitbart, 2012), an order of magnitude more abundant than bacteria (Fuhrman, 1999). Most ocean viruses prey upon bacteria (Fuhrman, 1999), and as a result, play a critical role in ecosystem dynamics. When these viruses (bacteriophage, or phage) lyse bacterial cells, carbon is converted to its dissolved form, slowing the export of carbon to the deep ocean (Suttle, 2005). Marine viruses thus ultimately influence biogeochemical cycling and can affect the rate of atmospheric warming (Wilhelm & Suttle, 1999; Danovaro et al., 2011). Besides being abundant and fundamental contributors to the Earth’s biogeochemical cycles, marine viruses are also extremely diverse. Although recent work examining viral protein clusters in metagenomes suggests the global virome may be smaller than previously thought, marine viruses still constitute one of the largest reservoirs of genetic diversity on the planet (Rohwer, 2003; Ignacio-Espinoza, Solonenko & Sullivan, 2013). Moreover, viruses can change the genetic makeup of bacteria through horizontal gene transfer (Lindell et al., 2004; Monier et al., 2009; Hurwitz & Sullivan, 2013) and affect bacterial community composition through lysis of specific host cells (Mühling et al., 2005). For all of these reasons, understanding the diversity of marine viruses has been a research focus for more than 20 years, since Bergh et al. (1989) documented high viral abundance in the oceans.

Studying viral diversity is challenging because the lack of a single gene common to all viruses precludes PCR-based surveys of total viral diversity (cf. 16S rDNA for bacteria) (Rohwer & Edwards, 2002; Dwivedi et al., 2012). However, a variety of signature genes exist that can be used to capture subsets of viral diversity (Adriaenssens & Cowan, 2014), such as the DNA polymerase gene for podophage (Breitbart, Miyake & Rohwer, 2004; Huang et al., 2010), capsid genes for myophage (Jameson et al., 2011; Chow & Fuhrman, 2012), and *psbA* for cyanophage (phage infecting cyanobacteria) (Chenard & Suttle, 2008). More recently introduced, *phoH* can capture viruses in multiple families of double-stranded DNA tailed phage (Goldsmith et al., 2011). *PhoH* has been found in phage that infect both heterotrophic and autotrophic hosts, including cyanobacteria such as *Prochlorococcus* (infected by phage P-SSM2 (Sullivan et al., 2005)), *Synechococcus* (infected by Syn9 (Weigele et al., 2007)), *Microcystis aeruginosa* (infected by Ma-LMM01 (Yoshida et al., 2008)), SAR11 bacteria (infected by pelagiphage HTVC008M (Zhao et al., 2013)), *Roseobacter* SIO67 (infected by SIO1 (Rohwer et al., 2000)), and at least eight *Vibrio* species (infected by KVP40 (Miller et al., 2003)), as well as autotrophic eukaryotes such as *Ostreococcus* and *Bathycoccus* (infected by OtV-1 and BpV1, respectively (Weynberg et al., 2009; Moreau et al., 2010)). Moreover, *phoH* is more likely to be present in marine phage than in phage originating from non-marine environments (Goldsmith et al., 2011).

The diversity of marine viral communities has been examined through numerous snapshots—analyses at a single time and place, or a depth profile studied at a single time. However, analysis of a surface viral community is unlikely to be representative of the viruses throughout the water column, and viral communities sampled in one season are likely to differ in composition from viruses at the same site but in a different season. Numerous studies have found that marine viral communities vary between depths and seasons (Bergh et al., 1989; Waterbury & Valois, 1993; Suttle & Chan, 1994; Wommack et al., 1999; Fuhrman, Griffith & Schwalbach, 2002; Riemann & Middelboe, 2002; Marston & Sallee, 2003; Wang & Chen, 2004; Mühling et al., 2005; Sandaa & Larsen, 2006; Payet & Suttle, 2008; Winget & Wommack, 2008), but multiyear experiments are needed to determine whether these patterns repeat over time.

This study improves current understanding of spatiotemporal dynamics of marine viral diversity by examining the Bermuda Atlantic Time-series Study (BATS) site in the northwestern Sargasso Sea. Ten years of monthly sampling at this site by Parsons et al. (2012) revealed annually recurring seasonal patterns of viral abundance in the upper 300 m of the water column. Viral abundance peaked every summer between 60 and 100 m depth concurrent with stratification of the water column. This subsurface peak in viral abundance was highly correlated with a localized increase in the concentrations of *Prochlorococcus*, the dominant photosynthetic organism at this site. Convective overturn each winter deepened the mixed layer and abolished the subsurface peak in viral abundance, leading to fairly stable viral concentrations in the upper water column (Parsons et al., 2012). Knowledge of the dynamics of viral abundance at the BATS site makes this site an ideal location to conduct a thorough analysis of dynamics in viral diversity. In this study, we performed 454 pyrosequencing of the *phoH* gene from the viral community in two different seasons (September = summer, stratified water column; March = winter, mixed water column) in multiple years over a depth profile from the surface to 1,000 m. To our knowledge this is the first examination of viral diversity using deep sequencing of a signature gene, allowing exhaustive sampling and determination of richness for a subset of the viral community. Both temporal and depth-related patterns of *phoH* composition emerge. Additionally, while the viral *phoH* community at BATS is very rich in terms of operational taxonomic units (OTUs; 97% sequence identity), the community is extremely uneven, with only a few OTUs dominating at many depths and times. The remainder of the viral community comprises OTUs that appear infrequently and have few members.

## Materials and Methods

### Sample collection and DNA extraction

Samples were collected from throughout a depth profile on September 2–3, 2008, March 9 and September 5, 2010, and March 28 and September 17, 2011. All samples were collected in the vicinity of the Bermuda Atlantic Time-series Study (BATS) site (31°40′N, 64°10′W) from 0, 20, 40, 60, 80, 100, 120, 140, 160, 180 (2008 and 2011 only), 200, 250 (2010 and 2011 only), 300, and 400 m depth. In 2008 and 2011, samples were also collected from 500, 600, 700, 800, 900, and 1,000 m. Whole seawater samples (100 mL) were filtered through a 0.22 µm Sterivex filter (Millipore, Billerica, MA) and then onto a 0.02 µm Anotop filter (Whatman, Piscataway, NJ). Anotop filters were stored at −80 °C until DNA was extracted with a MasterPure complete DNA and RNA purification kit (Epicentre Biotechnologies, Madison, WI) following the protocol of Culley & Steward (2007). Briefly, filters were defrosted, and all liquid was purged from the filter by pushing air through with a sterile syringe. A flame-sealed pipette tip was used to temporarily seal the filter outlet, and a mixture of 400 µL of 2X T&C lysis buffer (from the MasterPure kit) and 50 µg proteinase K was forced onto the filter. The filter was then incubated for 10 min in the air at 65 °C before the lysate was expelled into a microcentrifuge tube and immediately placed on ice. Then 150 µL of MPC protein precipitation reagent (from the MasterPure kit) was added to the lysate and vortexed vigorously for 10 s. The debris was pelleted by centrifugation at 10,000 × *g* for 10 min. Isopropanol was added to the recovered supernatant, and the tube was inverted 30 to 40 times. The DNA was then pelleted by centrifugation at 20,000 × *g* at 4 °C for 10 min and washed twice with 75% ethanol. Extracted DNA was resuspended in sterile water and stored at −20 °C.

### Collection of environmental data

Metadata associated with these sampling dates and depths are in Table S1 and are also available at the BATS website (bats.bios.edu) (Bermuda Institute of Ocean Sciences, 2014). The BATS monthly sampling is considered synoptic although it extends over three to five days (Michaels & Knap, 1996). All parameters used in this comparison (except chlorophyll *a* in March 2010) were collected within 36 h of each other to produce a composite profile for each sampling period. With the exception of September 2008, when all metadata was drawn from a BATS cruise conducted eight days after the cruise that provided the viral samples, temperature, salinity, and density measurements came from the same casts that provided the water from which viral DNA was extracted. Measurements of oxygen, chlorophyll *a* concentrations, and nutrients, as well as total bacterioplankton, *Prochlorococcus* and *Synechococcus* densities, were drawn from the same cruise where the viral samples were collected (except in September 2008) in order to establish biological and environmental context. No pigments were measured during the March 2010 cruise, so the chlorophyll *a* measurements for those samples were drawn from the previous BATS cruise that measured pigments, 12 days earlier.

### Amplification of the *phoH* gene

The extracted DNA was amplified in triplicate reactions using the strand displacement method of the Illustra GenomiPhi V2 DNA amplification kit (GE Healthcare, Piscataway, NJ) according to the manufacturer’s instructions and then pooled. Next a first-stage PCR was conducted for amplification of the *phoH* gene, using viral *phoH* primers vPhoHf (5′-TGCRGGWACAGGTAARACAT-3′) and vPhoHr (5′-TCRCCRCAGAAAAYMATTTT-3′) (Goldsmith et al., 2011). As shown by Goldsmith et al. (2011), these primers do not amplify known bacterial *phoH* genes. Since the publication of these primers, at least sixty new phage genomes that contain annotated *phoH* genes have been published in GenBank. Most of these phage infect heterotrophic bacteria. A phylogenetic tree (not shown) reveals that the primers would not capture the *phoH* genes from the newly-sequenced phage that infect heterotrophic bacteria, but with one exception (*Synechococcus* phage S-TIM5), all of the new cyanophage *phoH* sequences (*Synechococcus* phage S-MbCM100, S-MbCM7, S-SKS1, metaG-MbCM1, S-MbCM6, S-CAM1, Syn2, Syn10, KBS-M-1A, S-IOM18, S-CBM2, and S-SSM6a; *Prochlorococcus* phage P-SSM3, P-SS1, P-RSM3, and P-RSM6) fall into Group 2 of the phylogenetic groups identified in Goldsmith et al. (2011).

Four replicates of the PCR reaction were conducted for each sample, and the products were pooled after a reconditioning PCR and cleaning (see below). The 50-µL reaction mixture contained 1 U Apex *Taq* DNA polymerase (Genesee Scientific, San Diego, CA), 1X Apex *Taq* reaction buffer, 1.5 mM Apex MgCl_2_, a 0.5 µM concentration of each primer, 0.2 mM deoxynucleoside triphosphates, 0.08% bovine serum albumin, and 1 µL of template DNA (pooled GenomiPhi product). The reaction conditions were: (i) 5 min of initial denaturation at 95 °C; (ii) 35 cycles of 1 min of denaturation (95 °C), 1 min of annealing (53 °C), and 1 min of extension (72 °C); and (iii) 10 min of final extension at 72 °C.

Next, each PCR product underwent a reconditioning step as recommended by Berry et al. (2011), in order to minimize variation that can accompany different barcoded primers. The reaction mixture was the same as in the first-stage PCR, except that 10-bp barcodes were attached to the viral *phoH* primers (see Table S2). The template DNA consisted of 1 µL of product from the first-stage PCR reaction and the same reaction conditions were used, except that only 10 amplification cycles were run. After the reconditioning PCR, the four replicates for each sample were individually cleaned with a DNA Clean & Concentrator-25 kit (Zymo Research Corp., Irvine, CA) following the manufacturer’s instructions and resuspended in 45 µL of sterile water. The four replicates for each sample were pooled for quantification and downstream processing.

### DNA quantification and sequencing

The amount of DNA recovered for each sample was quantified using a real-time PCR measurement of fluorescence as suggested by Blotta et al. (2005), with Quant-iT PicoGreen as the detector (Life Technologies, Grand Island, NY). Each sample was run in duplicate, with the real-time PCR machine set to obtain a fluorescence reading during each of three 75-s cycles. The six fluorescence readings were averaged to obtain a mean fluorescence reading for each sample. After quantitation based on a standard curve, equal amounts of each sample (∼1600 ng) were placed into one of four pools for sequencing. Sequencing of the *phoH* amplicon was performed on the 454 GS FLX Titanium platform by Beckman Coulter Genomics (Danvers, MA). Before sequencing, Beckman ligated sequencing adaptors to each of the four pools, multiplexing them onto half of a picotiter plate. After sequencing, the four pools were de-multiplexed before the sequences were returned for analysis. The FASTA, .qual, and .sff files for each sample have been submitted to GenBank’s Sequence Read Archive as accession SRP039081. The BioProject Accession Number is PRJNA239691, and individual sample accession numbers are SAMN02670781 to SAMN02670865.

### Sequence analysis

After the barcodes were removed, the sequences were searched for the forward primer, and the downstream analyses proceeded with those sequences containing the forward primer. The sequences have been deposited in METAVIR (http://metavir-meb.univ-bpclermont.fr) under the public project name “Viral phoH at BATS—Goldsmith et al., 2014”, virome name “All phoH sequences, forward primer”. Mothur (Schloss et al., 2009) was used to align the sequences, trim them to include only the aligned space, filter out columns of the alignment that do not contain data, pre-cluster the sequences to merge sequences that are within two base pairs of a more abundant sequence, and remove chimeras, leaving 313,312 sequences. Using mothur, the sequences were grouped into operational taxonomic units (OTUs) defined by sequence identity of 97% or greater. Rarefaction curve data, Chao1 richness estimates, and inverse Simpson diversity estimates were also calculated using mothur, and plotted in R (R Development Core Team, 2013). In particular, the heatmap reflecting the inverse Simpson diversity estimates (Fig. S2) was plotted using the gplots (Warnes et al., 2009) and RColorBrewer (Neuwirth, 2011) packages in R. The heatmap reflecting the Chao1 richness estimates (Fig. S1) was plotted with the fossil package (Vavrek, 2011). Hierarchical clustering was performed with the average linkage method from a Bray-Curtis dissimilarity matrix using the picante package (Kembel et al., 2010). Distances were also computed using other algorithms (Euclidean, Manhattan, and Canberra; data not shown), but all methods produced the same general clustering trends. In order to bootstrap the dendrogram, Jaccard stability means were computed using the fpc package (Hennig, 2013). The dot plot (Fig. 5) was constructed with the lattice package (Sarkar, 2008).

*PhoH* sequences representative of each of 94 OTUs were selected for the phylogenetic tree: the 51 OTUs that contain at least 0.1% of the total number of sequences, and an additional 43 OTUs that contain at least 1% of the sequences from any individual sample. These 94 representative sequences have been deposited in GenBank’s Sequence Read Archive under accession SRP039081. The representative sequences are also in METAVIR (http://metavir-meb.univ-bpclermont.fr) under the public project name “Viral phoH at BATS—Goldsmith et al., 2014”, virome name “phoH OTU representatives”. Next, the HAXAT program (Lysholm, 2012) was applied to the sequences (against a custom-built database of viral *phoH* sequences) in order to correct homopolymer sequence errors (using default parameters, except that both strands were queried and a minimum score of 200 was used). *PhoH* sequences from several fully-sequenced phage genomes were added, and then an amino acid alignment was built from the sequences using MUSCLE (Edgar, 2004) with default parameters as implemented by TranslatorX (Abascal, Zardoya & Telford, 2010). Alignments based on amino acids rather than nucleotides are more accurate for protein-coding sequences such as *phoH* (Abascal, Zardoya & Telford, 2010). However, since nucleotide sequences better reflect the diversity of the *phoH* gene in the environment, the alignment was back-translated for phylogenetic analysis. FastTree (Price, Dehal & Arkin, 2010) was used to build an approximate maximum likelihood phylogenetic tree, with the Jukes-Cantor model of nucleotide evolution and the CAT approximation of a single rate of evolution across all sites. In R, the tree was prepared for aligning with the heatmap using the ape (Paradis, Claude & Strimmer, 2004) and phangorn (Schliep, 2011) packages. The heatmap (Fig. 6) was constructed and aligned with the tree using the gplots (Warnes et al., 2009), RColorBrewer (Neuwirth, 2011), and colorRamps (Keitt, 2012) packages. Permutational MANOVA analyses were conducted in PAST, version 3.01 (Anderson, 2001; Hammer, Harper & Ryan, 2001). Pairwise identities between OTU representative sequences were calculated using Sequence Demarcation Tool, version 1.2 (Muhire, Varsani & Martin, 2014).

### Mathematical modeling

A rank-abundance plot was constructed for each of the 85 samples in order to examine the mathematical distribution that best approximated the curve shape. Least-squares fits were determined for power law, exponential and lognormal shapes (Fig. S3; Supplemental Information 1) using the Solver package within Microsoft Excel. Based on the realization that each community was dominated by just a few OTUs, the interpolated median rank for each sample was also determined by considering the cumulative normalized abundance distribution and linearly interpolating to the rank value at which the cumulative fraction would reach 0.5. For examples of the interpolated median rank calculation, refer to Table S3.

## Results

**Figure 1:**
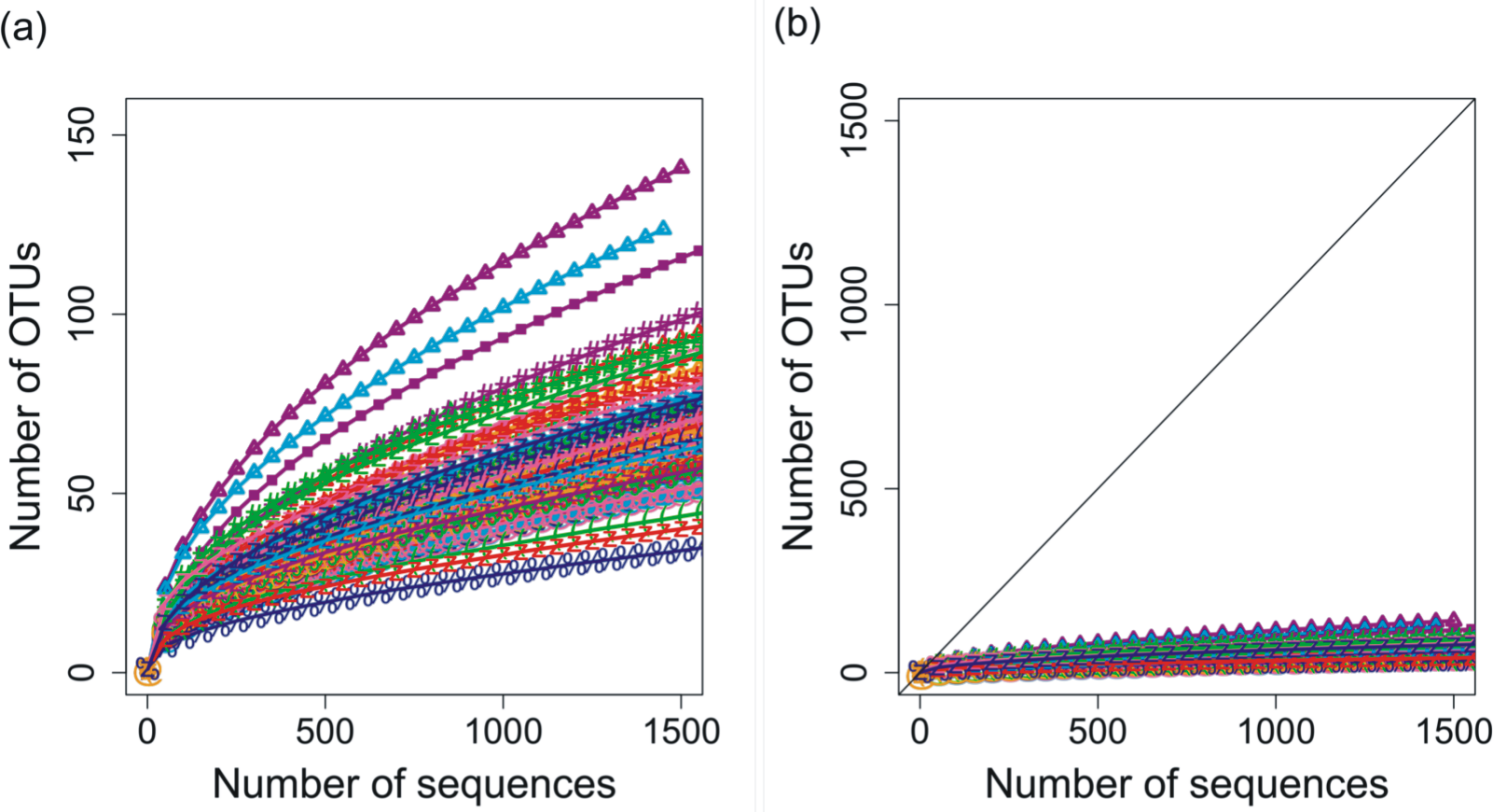
Rarefaction curves for *phoH* sequences from all 85 depths/times; OTUs are defined by sequence identity of 97% or greater. (A) Plotting with different scales on *x*- and *y*-axes demonstrates some separation of the rarefaction curves. Curve with greatest slope is September 2008, 0 m, while curve with least slope is March 2011, 1,000 m. (B) Plotting in relation to the 1:1 line demonstrates flattening of all rarefaction curves.

Deep 454 pyrosequencing of the *phoH* gene from 85 depth/time samples from the BATS site yielded a total of 313,312 sequences. The number of sequences per sample ranged from 288 to 12,791, with a median of 3,028 sequences recovered per sample. Based on operational taxonomic units (OTUs) defined by sequence identity greater than or equal to 97%, the total dataset consisted of 3,619 OTUs. Although the shape of the rarefaction curves differs for each of the 85 samples (Fig. 1A), the rarefaction curves for all of the samples have approached an asymptote (Fig. 1B), indicating that this level of sequencing sufficiently captured the diversity of the viral *phoH* gene at the BATS site.

**Figure 2:**
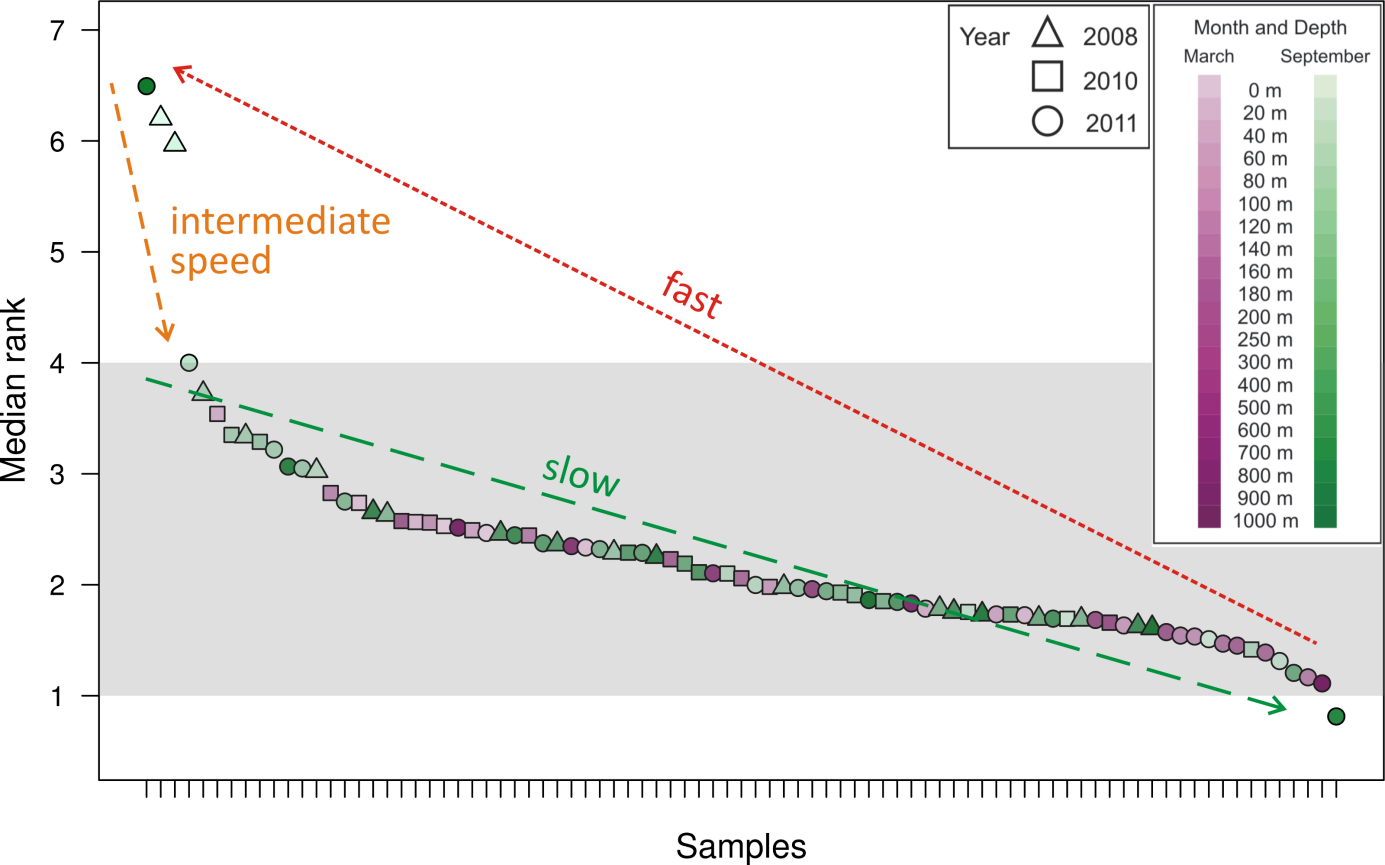
Median rank for each of the 85 communities sampled, arranged in descending order of median rank. Each ⋅ represents a sample and the median rank can be interpreted as the number of abundant OTUs in that community. Shading emphasizes that most of the communities have a median rank between 1 and 4. The outlier on the right, with a median rank less than one, is hypothesized to represent a community during a kill-the-winner scenario which leads to communities of the type represented by the outliers to the left with median rank between 6 and 7. Assuming that such kill-the-winner cycles are present in all the communities, the density of points in a region should be characteristic of the rate at which the community dynamics move through that region.

As a method of quantifying the number of highly abundant OTUs in the community, we introduced and explored the interpolated median rank parameter, which is the rank at which the cumulative distribution reaches 0.5. The median rank parameter therefore represents the number of OTUs in the top half of the community (Fig. 2). Excluding a few outliers, the interpolated median rank was between 1 and 4 OTUs. Even including the outlying samples—900 m in September 2011, 0 m and 20 m in September 2008 (high outliers), and 700 m in September 2011 (low outlier)–the range of values is quite narrow, remaining between 0.8 and 7 OTUs. This parameter demonstrates the highly uneven nature of the viral *phoH* communities, in which only a few OTUs represent more than half of any sample.

Calculation of two diversity metrics (Chao1 and the inverse Simpson’s index) did not reveal clear trends in viral *phoH* diversity over depth or time. The Chao1 richness estimator predicts the minimum richness of a community (Chao, 1984) and values for this dataset ranged from 89 to 1,164 *phoH* OTUs per sample. The richest *phoH* communities tended to be in the upper 300 m of the water column, with the exception of March 2011, when the 700 m community had the second highest richness in the depth profile (Fig. S1). Another diversity metric, the inverse Simpson’s index, incorporates not only richness but also a measure of evenness (Simpson, 1949); it is influenced by the abundance of the most common species (Magurran, 2004). The inverse Simpson’s index thus potentially provides greater insight and is more robust than diversity measures based solely on richness (Magurran, 2004). The inverse Simpson’s index ranges from a minimum of 1 (where only one OTU is present) to a maximum of the total number of OTUs (3,619 in this study) (Ricklefs & Lovette, 1999). According to the inverse Simpson’s index, the surface sample from September 2008 was the most diverse, with a diversity measure of 19.8, while the 700 m sample from September 2011 was the least diverse, with an inverse Simpson’s index of 2.5 (Fig. S2). The median value of the inverse Simpson’s index for all 85 samples was 6.3.

**Figure 3:**
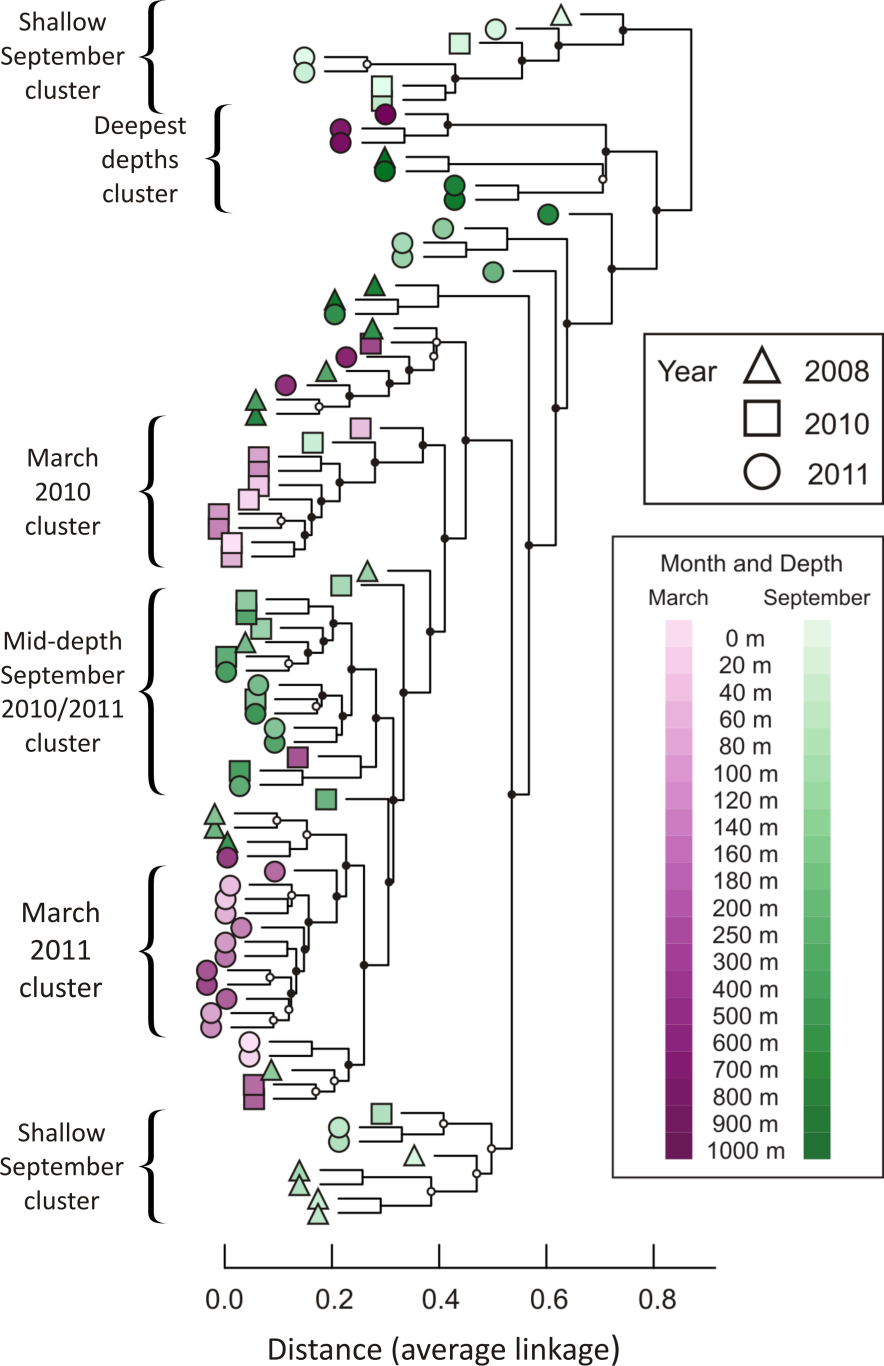
Dendrogram illustrating hierarchical clustering of all 85 depths/times. Samples are clustered using a Bray-Curtis dissimilarity matrix for all 3,619 OTUs. Nodes marked with a filled circle have a Jaccard stability mean greater than 75; nodes marked with an open circle have a Jaccard stability mean from 60 to 75 (Hennig, 2007; Hennig, 2008). Unmarked nodes have a Jaccard stability mean below 60.

A hierarchical cluster analysis performed after constructing a Bray-Curtis dissimilarity matrix revealed that similar depths and seasons cluster together (Fig. 3; dissimilarity matrix is presented in Table S4). For example, 14 of the 15 samples from September at depths shallower than 100 m fall into just two clusters, with no other samples contained in those clusters. In addition, 12 of the 16 samples collected in September 2010 and September 2011, from depths between 120 m and 500 m, form a well-supported cluster, with only two other samples contained in the cluster (March 2010, 300 m and September 2008, 180 m). Winter samples appear to cluster not only by season and depth, but also by year. The nine March 2010 samples from 0 m to 160 m are all in the same well-supported cluster, joined by only one other sample (September 2010, 40 m). Twelve samples from March 2011 form a well-supported cluster, including all depths from 40 m to 400 m. Regardless of season or year, deep water samples cluster together. Seven of the nine samples drawn from depths greater than or equal to 800 m form a well-supported cluster, which further divides into two subclusters according to season.

Over time, the *phoH* communities are more different between depths than they are within depths, according to a permutational MANOVA (*F* = 3.095, *p* = 0.0001). Pairwise comparisons reveal that many of the largest differences are between 1,000 m and other depths, especially depths shallower than 500 m (*F* values range from 4.7 to 14.3; *p*-values range from 0.015 to 0.03) (Table S5). Among the other depths investigated, 0 m is significantly different from every depth below 80 m (*F* values range from 3.13 to 5.79; *p*-values range from 0.008 to 0.04). The largest pairwise difference in *phoH* communities is between the 400 m community and the 1,000 m community (*F* = 14.3; *p* = 0.019). The depths with the fewest significant differences with other depths are 180 m (significantly different only from the 0 m community, *F* = 3.62, *p* = 0.037) and 500 m (significantly different only from the 0 m community (*F* = 4.06, *p* = 0.04) and the 40 m community (*F* = 3.77, *p* = 0.04)). Combining all depths and years, the season of sampling also influences the *phoH* viral community structure. The differences between the March and September *phoH* communities are greater than the differences within communities in the same month (*F* = 2.781, *p* = 0.011).

**Figure 4:**
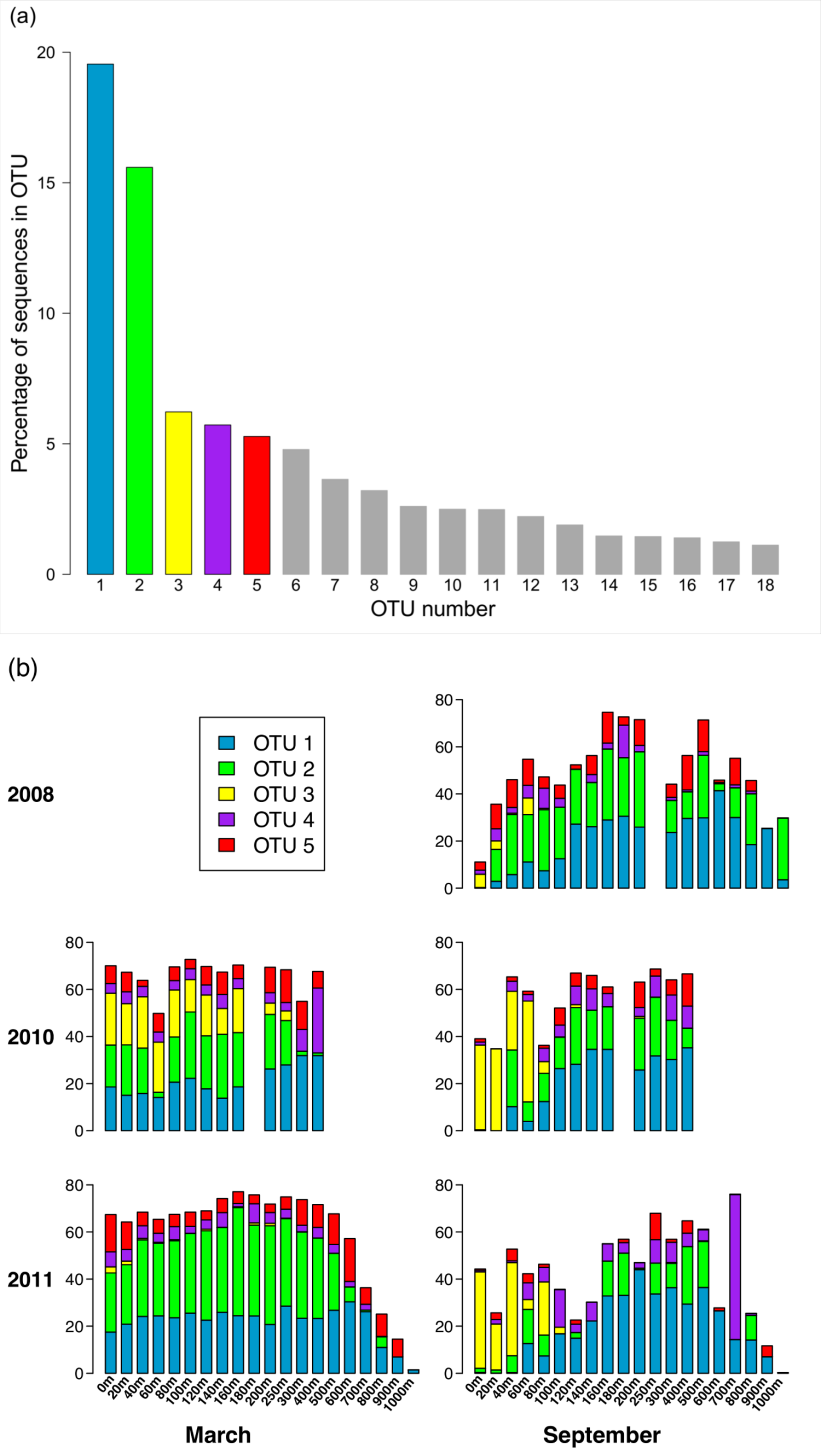
Composition of the *phoH* community according to the largest OTUs. (A) Percent of total sequences belonging to the 18 OTUs that contain at least 1% of the total sequences (*n* = 313, 312). (B) Percent of each sample belonging to OTUs 1 through 5. An empty spot indicates absence of sample for that date/depth.

Of the 3,619 OTUs recovered in this study, the vast majority of the OTUs were rare (∼96% of these OTUs contain <0.01% of the total number of sequences). Only 18 OTUs contain at least 1% of the total number of sequences (Fig. 4A). Fifty-one OTUs have at least 0.1% of the sequences, and 150 OTUs contain at least 0.01% of the sequences. Distribution of the sequences among the OTUs is highly skewed, in that together, the two largest OTUs (OTUs 1 and 2) contain more than one third of the sequences. The five largest OTUs (OTUs 1 through 5) contain 52.4% of the sequences, and more than 82% of the sequences are contained in the top 18 OTUs. Pairwise comparison of representative sequences from the five largest OTUs reveals that the two most similar pairs are OTUs 2 and 5 (94.6% identity) and OTUs 1 and 4 (74% identity). The remaining pairwise comparisons yield identities ranging from 59.5% to 65%.

**Figure 5:**
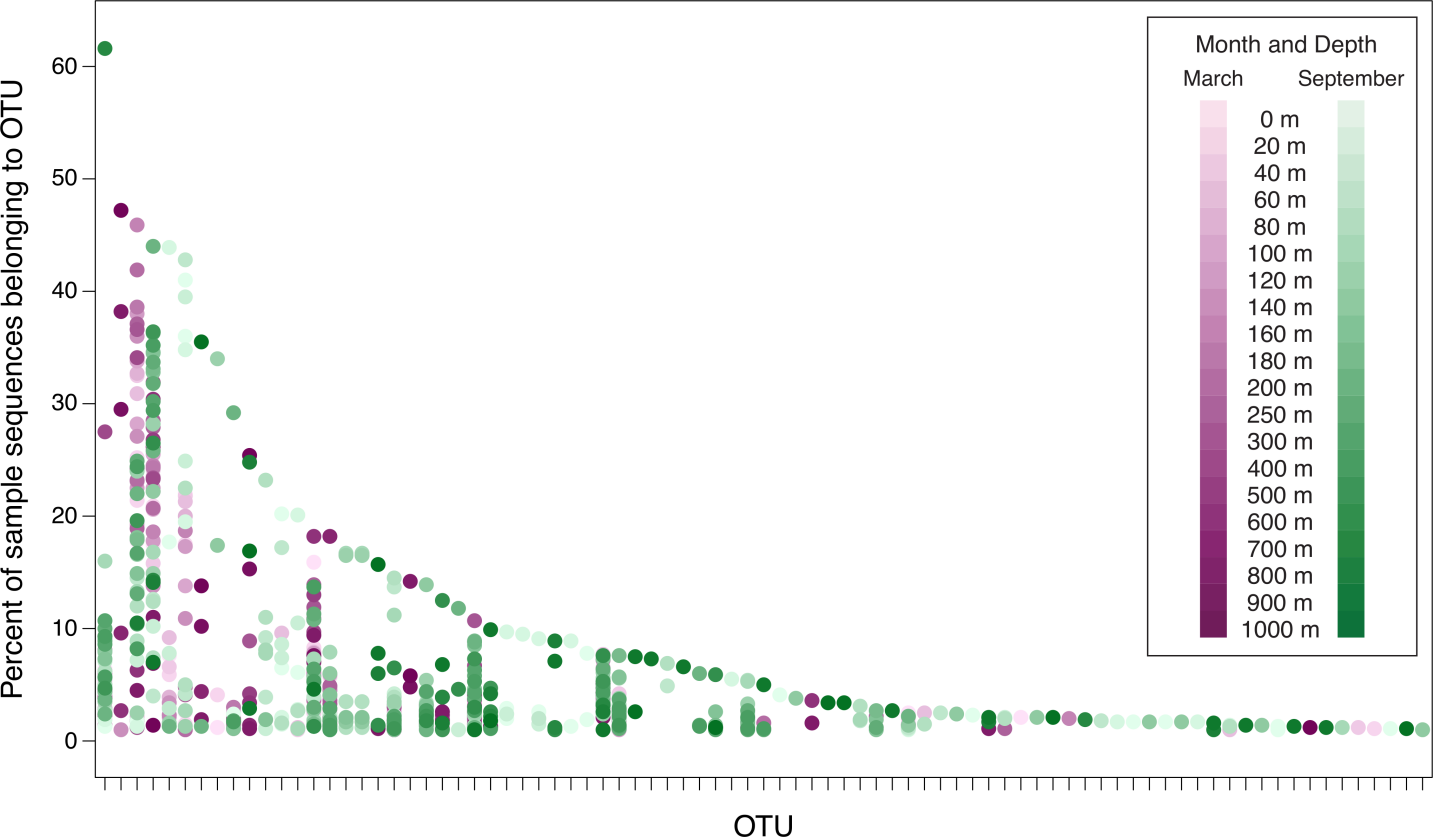
Percent of sample sequences falling in an OTU versus OTU. The dots represent samples and are color-coded to indicate month and depth. Samples from March 2010, September 2010, March 2011, and September 2011 are displayed in this plot. The September 2008 samples are not included in order to avoid overemphasizing seasonal differences (March 2008 was not sampled). The plot considers the 83 OTUs that contain ≥1% of the sequences from at least one sample from 2010 or 2011. Because the September 2008 samples are not included, the 83 OTUs displayed in this figure are a subset of the top 94 OTUs. No dots are displayed in OTU columns for samples in which less than 1% of the sample’s sequences belong to that OTU. OTUs are arranged along the *x*-axis in descending order of largest contribution to any single sample.

**Figure 6:**
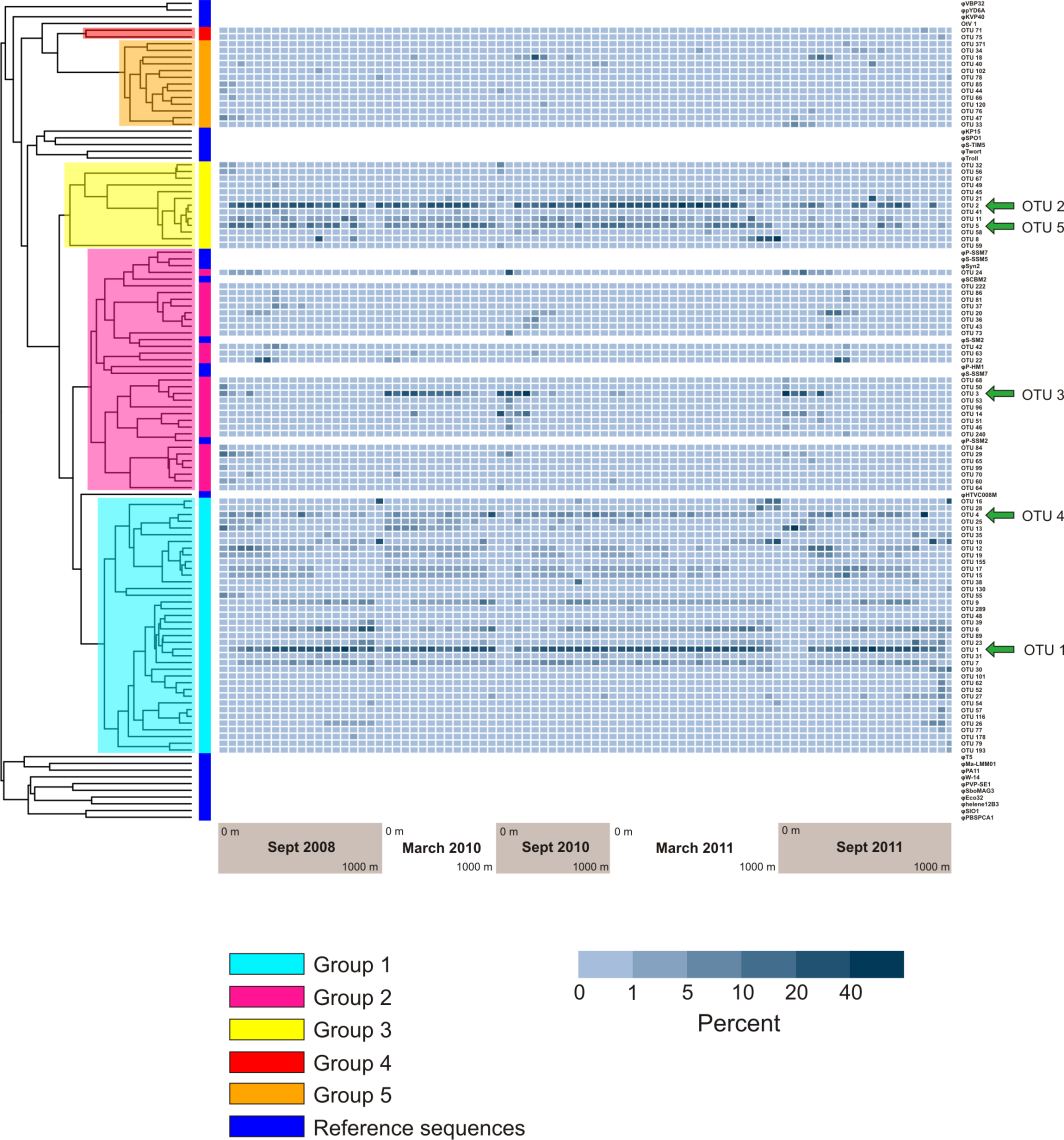
Prevalence of phylogenetically clustered OTUs in each sample, indicated as percent of each sample’s sequences that belong to each of the 94 top OTUs. Reference *phoH* sequences from fully-sequenced phage genomes (and one eukaryotic virus) are indicated with dark blue in the vertical color bar along the left side of the heatmap, between the heatmap and tree. The phylogenetic Groups 1–5 indicated in the tree are the same groups designated in Goldsmith et al. (2011).

Analysis of the five largest OTUs provides significant insight into compositional changes of the *phoH* community at BATS with season, depth, and year. Although the five largest OTUs together contain more than half of the total number of sequences, the degree to which those OTUs contribute to the community of each individual sample varies considerably (Fig. 4B). Sequences from these five OTUs comprise up to 77.1% of a sample (March 2011, 160 m) or as little as 0.2% of a sample (September 2011, 1,000 m). Although OTU 1 contains the largest proportion of sequences overall, this OTU is virtually absent from each of the three September surface communities. OTU 1 starts to appear in September below the surface, but sequences from OTU 1 do not reach 20% of the community until 100 m (2010), 120 m (2008), or 140 m depth (2011). In March, however, OTU 1 is a more consistent component of the *phoH* community throughout the depth profile; OTU 1 comprises 14% to 32% of the March 2010 community at all sampled depths, and 21% to 30% of the March 2011 community from the surface to 700 m.

Similarly, OTU 2 is a consistent presence in March 2011 from the surface to 500 m, and in March 2010 from the surface to 250 m (except for 60 m). However, OTU 2 constitutes less than 2% of each of the three September surface communities. OTU 2 becomes a larger portion of the September *phoH* communities starting with 20 m in September 2008 and 40 m in September 2010. In September 2011, OTU 2 has a sporadic and varied presence among the sampled depths. No OTU 2 sequences were present in the communities sampled at 140 m, 600 m, 900 m, and 1,000 m. At the other depths in September 2011, the contribution of OTU 2 sequences ranges from 0.01% at 100 m to 24% at 400 m.

OTU 3 has a strong presence in the upper 80 m during September 2010 and September 2011, as well as the upper 160 m of March 2010. OTU 3 appears in smaller percentages during September 2008 and March 2011. Sequences from OTU 3 are not found below 250 m, with a few exceptions where they constitute less than 1% of the *phoH* communities (300 m in March and September 2011, 400 m in September 2010 and March 2011, 500 m in September 2011). OTUs 4 and 5 constitute a smaller percentage of the *phoH* community at BATS; however, OTU 4 makes an especially large contribution to the 400 m community in March 2010 (27.5%) and the 700 m community in September 2011 (61.6%). The 61.6% contribution of OTU 4 to the September 2011 700 m community is the single largest contribution by any OTU to any sampled date and depth.

For the ease of data visualization, further analyses consider only 94 OTUs: the 51 OTUs that contain at least 0.1% of the total number of sequences, and an additional 43 OTUs that contain at least 1% of the sequences from any individual date/depth sample. Figure 5 demonstrates the percent of sequences in the top 94 OTUs from each of the samples from 2010 and 2011 (data underlying Fig. 5 are presented in Table S6). Few OTUs constitute a substantial portion of any individual sample. Only one OTU (OTU 4, discussed above) constitutes more than 50% of the sequences recovered from a single sample. Six OTUs constitute more than 40% of an individual sample. As the threshold decreases, more OTUs are included: 8 OTUs constitute at least 30% of a sample; 13 OTUs constitute at least 20%; and 24 constitute at least 10%. However, even at 5%, only 42 OTUs (out of a total of 3,619 OTUs identified in the dataset) meet the threshold. Thus only 1.1% of the OTUs constitute at least 5% of any sample, and the vast majority of the OTUs are rare. Figure 5 also demonstrates the seasonal nature of some OTUs. Some OTUs appear only in *phoH* communities sampled in March, while other OTUs appear only in September samples.

Figure 6 displays a phylogenetic tree of the *phoH* gene containing representatives from each of the top 94 OTUs, as well as the *phoH* gene from several fully-sequenced “reference” viral genomes. The heat map next to the tree displays the prevalence of each OTU (rows) in each sample (columns). The groups identified in the phylogenetic tree are the same groups identified in a previous study of marine viral *phoH* diversity (Goldsmith et al., 2011). Despite the greatly increased sequencing depth in the present study, no new phylogenetic groups were identified among those top 94 OTUs. However, some of the rare OTUs (those containing less than 0.1% of the total number of sequences and less than 1% of the sequences from any individual sample) fall outside the previously-defined groups (data not shown). The five largest OTUs (Fig. 4) belong to three phylogenetic groups: OTUs 1 and 4 are in Group 1; OTUs 2 and 5 are in Group 3; and OTU 3 is in Group 2. Groups 2 and 3 contain known cyanophage *phoH* sequences; however, hosts for phage in the other groups are currently unknown.

**Figure 7:**
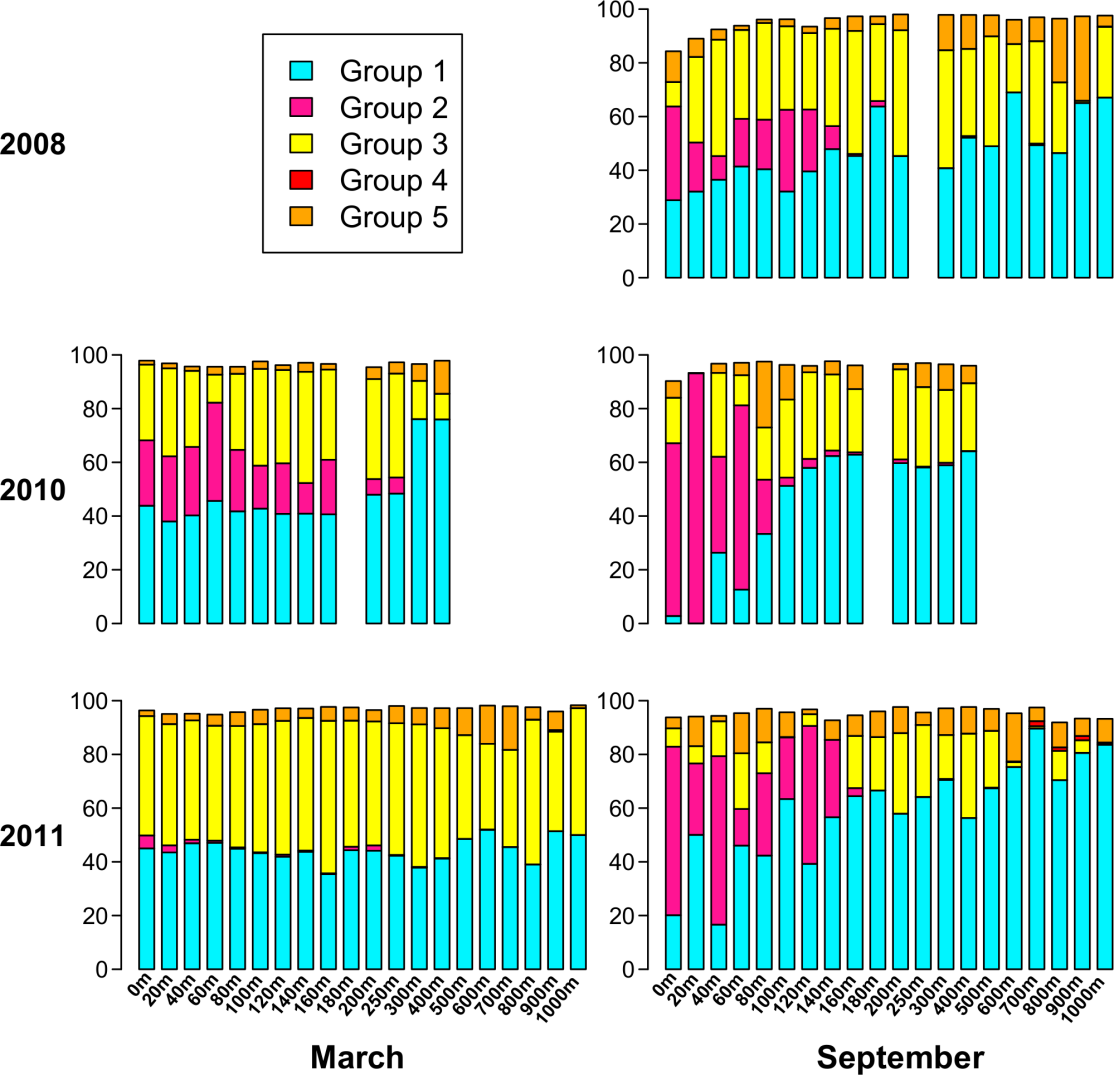
Percent of each sample belonging to phylogenetic Groups 1 through 5. An empty spot indicates absence of sample for that date/depth.

Based on the phylogenetic groups to which each of the top 94 OTUs belongs (Fig. 6), Fig. 7 displays the phylogenetic group composition of each sample. Group 1 (containing OTUs 1 and 4) is a dominating presence throughout the dataset, constituting at least 40% of 68 of the 85 samples, and at least 30% of 78 of the samples. Group 2, containing OTU 3, is limited to the upper part of the water column. While Group 2 appeared in each summer *phoH* community, no consistent pattern emerged for Group 2 in winter. It is virtually absent in March 2011, but represents from 11% to 36% of the viral communities in March 2010 from the surface to 160 m. Group 3 (containing OTUs 2 and 5) has a strong presence at all depths in March 2011, but is more varied in its abundance throughout the depth profile in March 2010. Group 3 comprises a smaller portion of the three September surface communities than it does of the March surface communities. In September 2011 in particular, Group 3 forms less than 15% of every sample from the surface through 140 m, with the exception of the 60 m community (20.7%).

Overall, Groups 4 and 5 comprise a smaller part of the sampled *phoH* communities, and are more represented at depth than in the upper water column. In only 3 out of 85 samples did Group 4 comprise at least 1% of the *phoH* community. All three of those samples were from September 2011, at depths of 700 m, 800 m, and 900 m. Group 5 is more prevalent than Group 4, but even so, only five samples contain Group 5 as at least 15% of the community. The maximum contribution Group 5 makes to a sample is in September 2008, 900 m, where it constitutes 31% of the *phoH* community. However, no patterns emerge in the environmental metadata to explain the unusual abundances of Groups 4 and 5 in these samples.

## Discussion

The present study demonstrates statistically significant temporal patterns in viral diversity. A permutational MANOVA and a dendrogram based on a Bray-Curtis dissimilarity matrix show that the month of sampling significantly influences composition of the *phoH* viral community (Table S5), and that when considered individually, similar depths and seasons tend to cluster together (Fig. 3 and Table S4). These data are consistent with numerous previous studies that have demonstrated temporal variation in marine viral communities using vastly different methods including pulsed field gel electrophoresis (PFGE) randomly amplified polymorphic DNA (RAPD) PCR, denaturing gradient gel electrophoresis (DGGE) and fluorescence intensity (Wommack et al., 1999; Steward, Montiel & Azam, 2000; Mühling et al., 2005; Sandaa & Larsen, 2006; Winget & Wommack, 2008; Winter, Kerros & Weinbauer, 2009; Jamindar et al., 2012; Magiopoulos & Pitta, 2012). Based on more than a year of monthly analyses, two signature genes in cyanomyophage (*g20* and *psbA*) showed clear distinctions between the summer viral community and the fall/winter viral community in coastal California (Clasen et al., 2013) and analysis of myophage isolates through the *g20* signature gene also revealed seasonal variations of abundance and diversity in coastal Rhode Island waters (Marston & Sallee, 2003).

Most of these studies have been limited to temporal analyses within a single year, so the repeatability of these patterns cannot be addressed. Multiyear time-series studies, such as the data presented here, are especially valuable for addressing this issue. The cyanopodophage community of the Chesapeake Bay, analyzed via the DNA polymerase gene during winter and summer for two years, exhibited repeating seasonal differences, and winter phage communities sampled in different years grouped more closely with each other than with summer phage communities from the same year (Chen et al., 2009). For cyanophage isolated from Narragansett Bay, similarity analysis based on the *g43* DNA polymerase gene showed clustering according to season, such that the cyanomyophage community composition was more similar between samples from the same season of different years than between samples from a different season in the same year (Marston et al., 2013). Viral communities at the site of the San Pedro Ocean Time-series (SPOT) also displayed seasonally recurring patterns of diversity as measured by T-RFLP analysis of the *g23* gene (Chow & Fuhrman, 2012). Communities 3–7 months apart were negatively correlated, while communities from adjacent months were highly correlated, as were communities from the same month one year apart (Chow & Fuhrman, 2012). Using the same type of analysis, Pagarete et al. (2013) studied changes in the myophage community sampled monthly for two years from water in Raunefjorden, Norway and observed three distinct viral communities depending on the season: summer, fall, and winter/spring.

The present study found that most of the sequences are in a few (large) OTUs that were common (i.e., found in a high proportion of sampling dates/depths). The remaining OTUs (the bulk of the OTUs) were small and rare. It should be noted that the methods used in this study are subject to potential biases. The whole genome amplification method used here, multiple displacement amplification (MDA), relies on random hexamers and the activity of the phi29 polymerase (Dean et al., 2002). Community composition of a population amplified by MDA is skewed (Pinard et al., 2006; Yilmaz, Allgaier & Hugenholtz, 2010), with a bias against templates of high GC content (Bodelier et al., 2009; Yilmaz, Allgaier & Hugenholtz, 2010) and in favor of circular genomes (Kim & Bae, 2011). In addition, the primers used in this study, designed from three cyanophage and one vibriophage, will only amplify a limited subset of the *phoH* genes known to occur in phage and may not amplify *phoH* genes from phage of heterotrophic bacteria (Goldsmith et al., 2011). This study applied barcoded versions of the *phoH* primers, but used them in a reconditioning step in order to reduce variation that can result from differences in the barcodes (Berry et al., 2011) (see Materials and Methods). Finally, PCR is always subject to bias because different templates amplify with different efficiencies (Polz & Cavanaugh, 1998; Lee et al., 2012) and because in later cycles of the reaction, the increased number of templates results in a greater chance of templates annealing to each other rather than to primers (Suzuki & Giovannoni, 1996). Despite these potential biases, because the same methods were used to process all samples in this study, between-sample comparisons are appropriate, and the outcomes of this study are consistent with numerous previously published studies.

The present results are in concordance with the findings of a 78-day time series in which most of the OTUs appeared in less than 25% of the samples, but more than 80% of the viral community consisted of OTUs that appeared in at least 90% of the samples (Needham et al., 2013). A culture-based study by Marston et al. (2013) obtained similar results for cyanophage isolates. At one location, the 12 most abundant OTUs (out of 108) represented 63.5% of the isolates. At other locations, the five most abundant OTUs (out of 162) represented 58% of the isolates (Marston et al., 2013). In another study, monthly sampling over two years showed that the most commonly observed OTUs had higher average and maximum contributions to the viral community (based on the *g23* gene), while the OTUs that appeared less frequently in the samples tended to represent fewer sequences from the viral community (Pagarete et al., 2013). Analysis of the *g23* gene in another study yielded concordant findings: over three years of monthly samples, the most common OTUs made up a higher proportion of the viral community than did the least common OTUs (Chow & Fuhrman, 2012).

Both the present study and Parsons et al. (2012) underscore the importance of investigating both time and depth in order to understand the dynamics of a marine viral community. In this study, the OTU composition of the upper 200 m is fairly consistent in March when the water column at BATS is well-mixed (Fig. 4B), while the September samples, collected during summer stratification at BATS, reflect a much more variable composition of the *phoH* community in the upper 200 m. Part of the September variability in OTU composition is due to the presence of OTUs belonging to phylogenetic Group 2 (Fig. 7). Group 2, which contains the vast majority of *phoH* sequences from phage of *Prochlorococcus* and *Synechococcus* (Fig. 6), is abundant in the upper water column but absent from deeper depths, suggesting that this group is dominated by *phoH* genes of cyanophage. The 20 m sample from September 2010 is especially noteworthy, because more than 93% of the community belongs to phylogenetic Group 2 (Fig. 7). The high abundances of Group 2 *phoH* sequences in the viral communities from the surface to 60 m in September 2010 correspond to elevated average abundances of *Prochlorococcus* at those depths in September 2010 compared to the same depths from September 2008 and September 2011 (Parsons et al., 2012; Bermuda Institute of Ocean Sciences, 2014).

In winter, when the water column is well-mixed, Group 2 exhibits much greater interannual variation. In March 2010, Group 2 forms between 11% and 36% of each sample from 0 m to 160 m. However, in March 2011, Group 2 is virtually absent from the depth profile. At every depth from the surface to 160 m, the abundance of *Prochlorococcus* was higher in March 2010 than in March 2011, and the water temperature was also higher (Table S1). The differences between *Synechococcus* abundances at these depths between March 2010 and March 2011 were more variable, but the average *Synechococcus* abundance from the surface to 160 m was 4% higher in March 2010 than in March 2011 (Table S1). The similarity of the trends in Group 2 *phoH* abundance and cyanobacterial abundance, along with the concentration of Group 2 *phoH* genes in the photic zone, supports the hypothesis that the Group 2 *phoH* sequences belong to cyanophage. Assuming that Group 2 does in fact represent cyanophage, these data are consistent with a study by Wilson et al. (1999) showing that the cyanophage population structure was similar throughout the upper 100 m at a well-mixed Atlantic Ocean site, while at another site, where the water column was stratified, the structure of the cyanophage population was variable throughout the depth profile.

Persistence of some OTUs and transience of other OTUs are recurring themes in studies of viral diversity, and the present study is no exception. At SPOT, certain OTUs showed repeating seasonal patterns, but the patterns varied among OTUs: some OTUs persisted throughout the year at moderate levels, while others had peak abundances in a particular season (Chow & Fuhrman, 2012). In a hypersaline lake in Australia, over nearly three years, much of the viral community was dynamic, while at least one assembled viral genome and two other viral genome fragments appeared in 91% to 100% of the samples (Emerson et al., 2012). In Lake Ontario, quantitative PCR (qPCR) was used to track the abundance of three algal virus genes for 13 months (Short & Short, 2009). Two of the genes appeared in nearly every sample, with seasonal variations in abundance, while the other gene appeared in only a few samples but at higher abundance than the other two genes. This study posited that some aquatic viruses persist throughout the year, while others are transient. Rozon & Short (2013) expanded upon the results of that study by using qPCR to monitor the abundance of 10 viral genes at three stations in an embayment of Lake Ontario from May to October. The genes (from algal viruses and freshwater cyanophage) exhibited several different patterns of abundance. Some OTUs appeared at all locations and all time points at fairly constant abundances, some taxa appeared at all locations but only sporadically, and other taxa showed patchy distribution (Rozon & Short, 2013). Similarly, in the present study, we find that some OTUs persist throughout the seasons, depths, and years, while many other OTUs make sporadic appearances at just one or a few times or depths.

Along with all other available studies, the present data support the Bank model, where marine viral communities contain a small subset of abundant viruses and a large bank fraction of rare viruses (Breitbart & Rohwer, 2005). However, in contrast to the originally proposed Bank model, where viruses cycle between the abundant and bank compartments, this study demonstrates dynamics similar to those observed by Rodriguez-Brito et al. (2010), where the largest *phoH* OTUs persist throughout changes in seasons, depths, and years. The appropriateness of the Bank model is supported by the interpolated median rank values, which represent the number of OTUs in the top half of the population and can roughly be interpreted as the number of abundant OTUs. Most of the samples, representing the *phoH*-containing viral communities at a particular snapshot in space and time, have between 1 and 4 abundant members as reflected in the interpolated median rank (Fig. 2). The outliers, clearly visible in Fig. 2, point to a possible signature of kill-the-winner dynamics (Thingstad & Lignell, 1997). In the scenario that gives such dynamics its name, a community with a highly active bacterial strain (the winner) is subject to a bloom of phage that specifically infects it, reducing the abundance of the winning strain and releasing carbon and nutrients for consumption by scavenger members of the bacterial community. For each community containing a median rank of one or less (one clear winner), many communities with a median rank of several competitors vying for the vacated top spot would be expected, as scavenger “rare” bacterial taxa move temporarily from the bank to the abundant class for as long as the available substrates from the lysis event last. At the completion of this relatively brief period, the median rank (i.e., size of the abundant group) moves back to the stable 1–4 member region.

The above line of reasoning leads to the expectation of cycles in the number of abundant members as a consequence of kill-the-winner dynamics between phage-host pairs. We conjecture that the 85 samples analyzed here were taken at random times along such a cycle in median rank and thus the number of sampled points in a median rank interval is indicative of the fraction of the time that the dynamics of such a community spends in that median rank range. The outliers (both high and low) in Fig. 2 are then interpreted as evidence of such cycles. These data therefore provide insight into the relative time scales involved in such a cycle: a slow drift of one of the abundant OTUs to dominance, followed by a rapid decline of this dominant OTU to many abundant OTUs (6 or 7) for a brief period, after which we again establish a relatively long-lasting abundant group size of 1–4. This scenario is illustrated by the arrows in Fig. 2, which demonstrate the cyclical dynamics that consist of the following steps: (1) drift of abundant group size from 4 to 1 (slowest step); (2) jump of the abundant group size from 1 to 6–7 (fastest step); (3) drift back to steady state from 7 to 4 (intermediate speed step). The distinctness and relative speed of these three regimes have been generally conjectured for pelagic phage-host dynamics and are the chief features of canard dynamics, which are cycling dynamics that experience very different speeds (of traversing the cycle) during different stretches along the cycle (Gavin et al., 2006; Hoffmann et al., 2007). If each of the sampled communities is considered a random selection from these trajectories (Hoffmann et al., 2007), the fact that only four outlying snapshots were captured in steps (2) and (3) (i.e., outside the typical abundant group of 1–4 members) suggests that these stages comprise approximately 5% of the cycle time.

Our findings modify the Bank model by suggesting that exchange rarely occurs between compartments, and by analyzing the median rank of these communities to demonstrate the relative timescales on which kill-the-winner interactions occur. The *in situ* viral *phoH* diversity data generated in the present study are consistent with the constant-diversity dynamics model proposed by Rodriguez-Valera et al. (2009) and theoretical analyses by Thingstad et al. (2014), suggestive of the strong role of phage predation in driving bacterial diversification among successful lineages in the environment. Moreover, these data are consistent with the findings of Rodriguez-Brito et al. (2010), who studied virus and host dynamics in four aquatic environments and demonstrated the persistence of broad viral and host taxa concurrent with kill-the-winner-type fluctuations at the level of host strains and viral genotypes. Together, these experimental and theoretical findings support the hypothesis that a given sample in the ocean contains a small number of highly successful abundant viruses (which comprise the top 50% of community abundance), complemented by a large number of rare bank species. Transient conditions related to major lysis events occasionally (approximately 5% of the snapshots captured in this study) enable OTUs in the bank to become abundant, but the system rapidly returns to steady state.

## Conclusion

This study represents the first deep sequencing of a signature gene to explore marine viral diversity. Using the viral *phoH* gene to examine the composition of the viral community in the Sargasso Sea, patterns of diversity emerged related to both depth and time. Moreover, the study confirmed the findings of previous research in determining that some viral OTUs persist in the environment over depth and time, while many other OTUs appear only sporadically. These data conform to one aspect of the Bank model, in that the abundant OTUs constitute a small portion of the total number of viral OTUs, while most of the viral OTUs are rare. However, these data modify the Bank model by suggesting that rare OTUs stay rare, rather than cycling between the abundant and rare fractions over time.

## Supplemental Information

10.7717/peerj.997/supp-1 Figure S1 Heatmap displaying Chao1 minimum richness estimate for all dates and depths A black bar indicates absence of sample for that date/depth.

10.7717/peerj.997/supp-2 Figure S2 Heatmap displaying inverse Simpson index diversity estimate for all dates and depths A black bar indicates absence of sample for that date/depth.

10.7717/peerj.997/supp-3 Figure S3 Equations for least-squares fits determined for power law, exponential and lognormal shapes Equations are accompanied by the fits shown for a sample rank-abundance graph.

10.7717/peerj.997/supp-4 Figure S4 Sum of squared errors used as the goodness of fit criterion for selecting parameter values of the three functional forms used for the rank-abundance curves The three models fit about equally well, with the lognormal model having the lowest sum of squared errors more often than the other two models.

10.7717/peerj.997/supp-5 Figure S5 Correlation between the parameters responsible for the rate of decrease in the rank-abundance curve fits: the power law parameter b and the exponential rate parameter k

10.7717/peerj.997/supp-6 Table S1 Sample collection data, environmental conditions and median rank for each sample in this study BDL, below detection limit; n.d., no data.

10.7717/peerj.997/supp-7 Table S2 Barcodes used to tag *phoH* amplicons

10.7717/peerj.997/supp-8 Table S3 Examples of calculation of interpolated median rank

10.7717/peerj.997/supp-9 Table S4 Bray-Curtis dissimilarities from which the clustering dendrogram in Fig. 3 was computed

10.7717/peerj.997/supp-10 Table S5 F values for pairwise comparisons of depths by permutational MANOVA Input consisted of normalized abundance data for all depths/times and all 3,619 OTUs. Highlighted cells reflect *F* values for which the *p*-value is <0.05.

10.7717/peerj.997/supp-11 Table S6 Data underlying Fig. 5, consisting of the percentage of a sample’s sequences that each of the top OTUs contributes to each sample in 2010 and 2011

10.7717/peerj.997/supp-12 Supplemental Information 1 Discussion of distribution of fits to rank abundance curves

## Additional Information and Declarations

### DNA Deposition

The following information was supplied regarding the deposition of DNA sequences: The FASTA, .qual, and .sff files for each sample have been submitted to GenBank’s Sequence Read Archive as accession SRP039081. The BioProject Accession Number is PRJNA239691, and individual sample accession numbers are SAMN02670781 to SAMN02670865. The sequences have also been deposited in METAVIR (http://metavir-meb.univ-bpclermont.fr) under the public project name “Viral phoH at BATS—Goldsmith et al., 2014”, virome name “All phoH sequences, forward primer.” The 94 representative OTU sequences have been deposited in GenBank’s Sequence Read Archive under accession SRP039081. The representative sequences are also in METAVIR (http://metavir-meb.univ-bpclermont.fr) under the public project name “Viral phoH at BATS—Goldsmith et al., 2014”, virome name “phoH OTU representatives.”
