# Supplementary figures and images for: Deep sequencing of the viral phoH gene reveals temporal variation, depth-specific composition, and persistent dominance of the same viral phoH genes in the Sargasso Sea
Source: PeerJ. 2015 Jun 16;3:e997. doi: 10.7717/peerj.997 (PMC4476143; doi:10.7717/peerj.997)

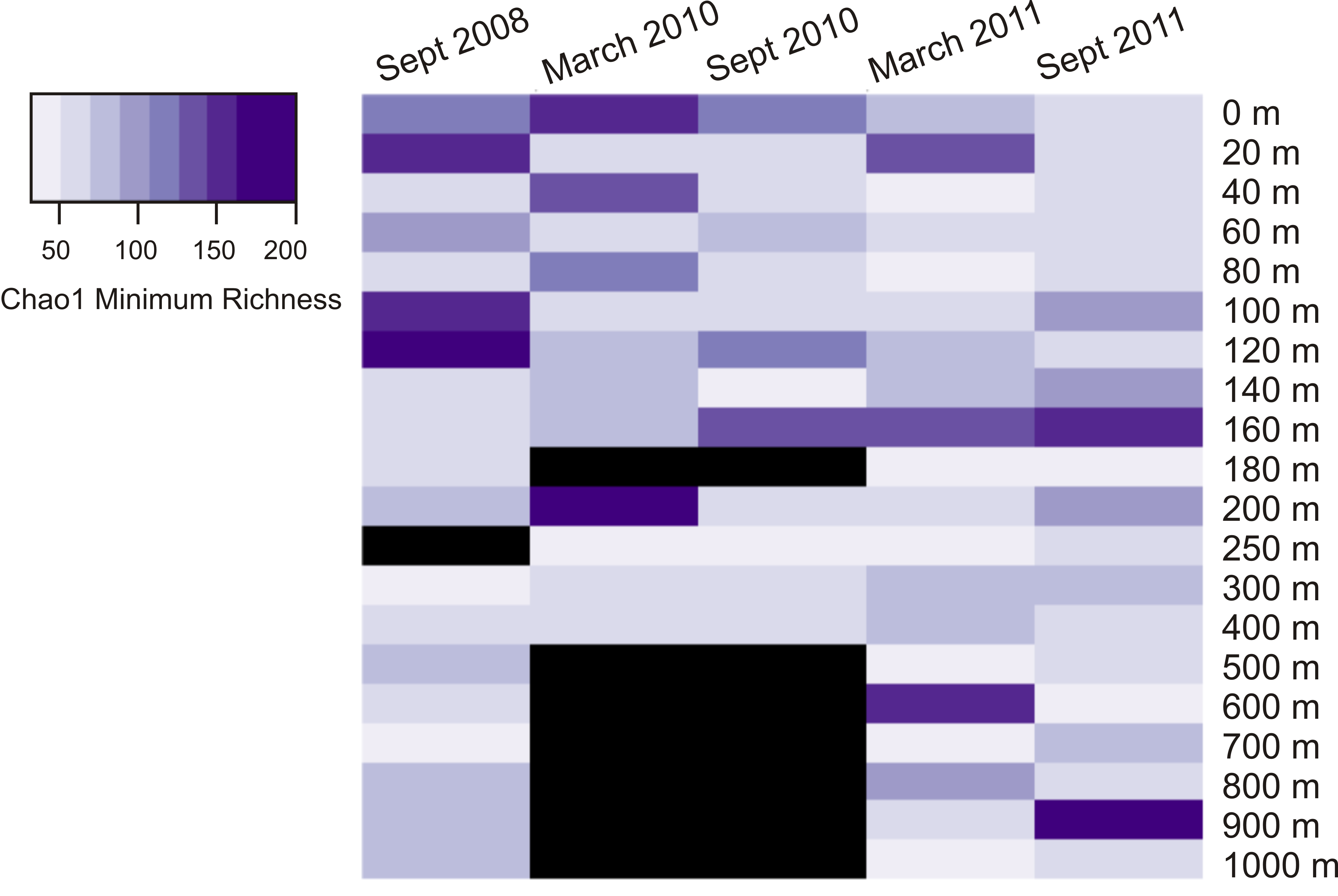

Supplement: Figure S1 — A black bar indicates absence of sample for that date/depth. [file peerj-03-997-s001.png]

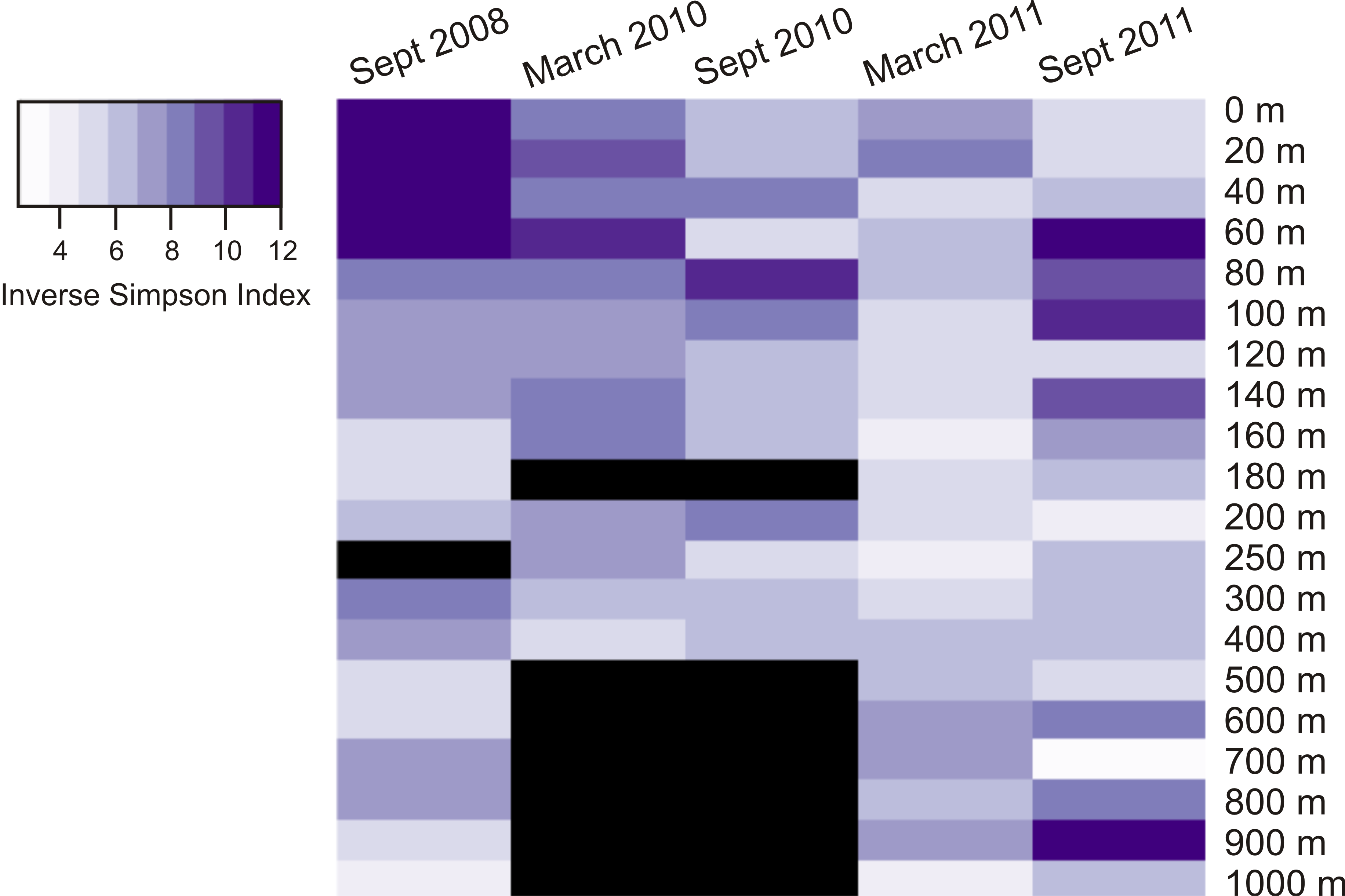

Supplement: Figure S2 — A black bar indicates absence of sample for that date/depth. [file peerj-03-997-s002.png]

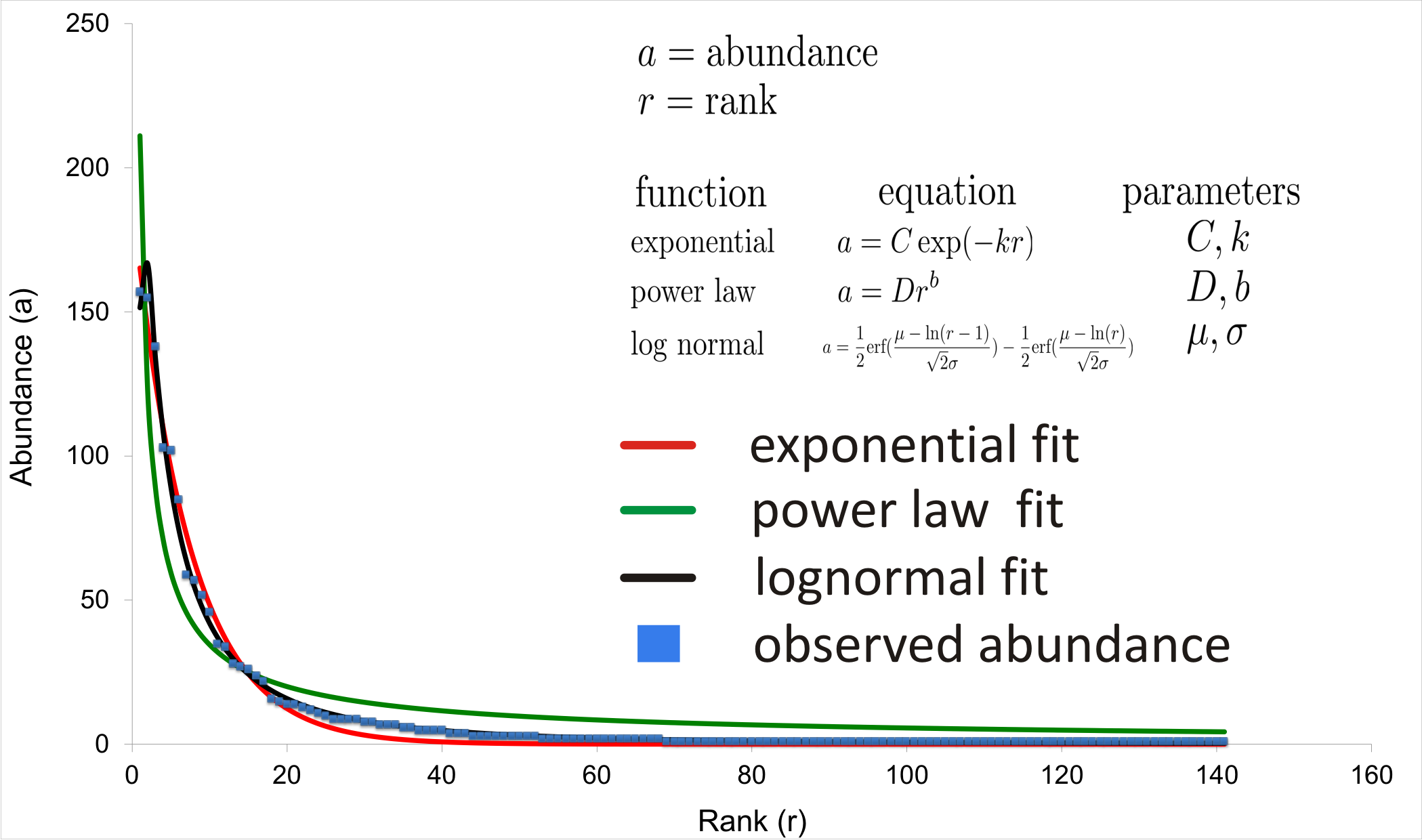

Supplement: Figure S3 — Equations are accompanied by the fits shown for a sample rank-abundance graph. [file peerj-03-997-s003.png]

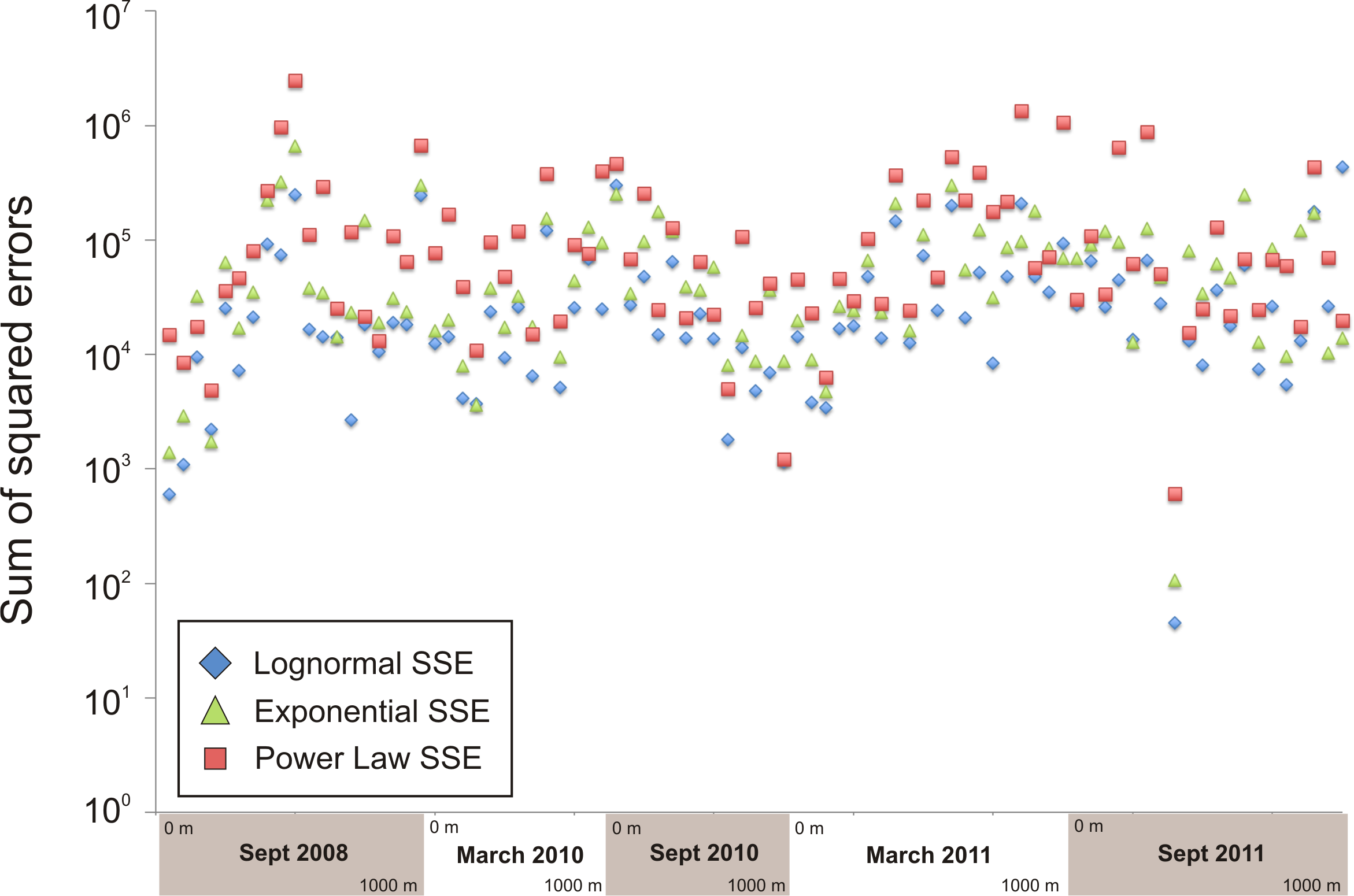

Supplement: Figure S4 — The three models fit about equally well, with the lognormal model having the lowest sum of squared errors more often than the other two models. [file peerj-03-997-s004.png]

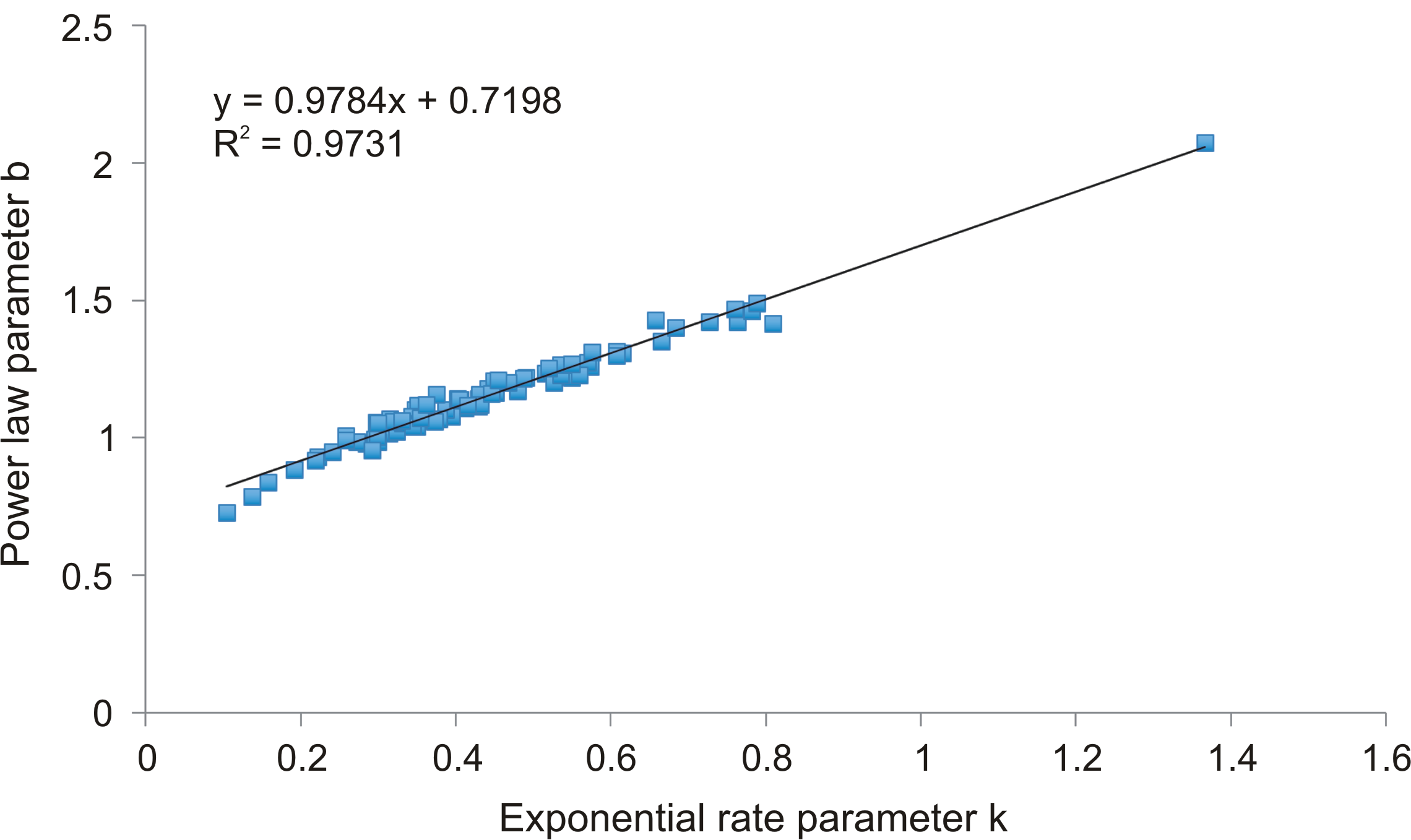

Supplement: Figure S5 [file peerj-03-997-s005.png]
