# Supplementary material for: Deep sequencing of the viral phoH gene reveals temporal variation, depth-specific composition, and persistent dominance of the same viral phoH genes in the Sargasso Sea
Source: PeerJ. 2015 Jun 16;3:e997. doi: 10.7717/peerj.997 (PMC4476143; doi:10.7717/peerj.997)
Supplement: Table S1 — BDL, below detection limit; n.d., no data. [file peerj-03-997-s006.docx]

| Year | Month | Depth  (m) | Median rank | Temperature  (ºC) | Salinity  (psu) | Density  (sigma-t, kg m^-3^) | Oxygen  (µmol  kg^-1^) | Nitrate (µmol  kg^-1^) | Nitrite (µmol kg^-1^) | Phosphate (µmol  kg^-1^) | Bacterial abundance  (x 10^8^ mL^-1^) | | *Prochlorococcus*  (x 10^4^ mL^-1^) | *Synechococcus*  (x 10^3^ mL^-1^) | Chlorophyll *a*  (ng kg^-1^) |
| --- | --- | --- | --- | --- | --- | --- | --- | --- | --- | --- | --- | --- | --- | --- | --- |
| 2008 | September | 0 | 6.20 | 28.602 | 36.291 | 23.17 | 198.7 | 0.06 | BDL | BDL | | 3.5 | 21458 | 7027 | 16 |
|  |  | 20 | 5.97 | 27.645 | 36.471 | 23.622 | 208.6 | 0.06 | BDL | BDL | | 4.2 | 18904 | 8356 | 17 |
|  |  | 40 | 3.03 | 24.058 | 36.597 | 24.838 | 225.4 | 0.06 | BDL | BDL | | 4.3 | 24807 | 3909 | n.d. |
|  |  | 60 | 3.72 | 21.671 | 36.708 | 25.614 | 225.7 | 0.06 | BDL | BDL | | 4.1 | 37808 | 5190 | n.d. |
|  |  | 80 | 3.34 | 20.161 | 36.626 | 25.965 | 220.1 | 0.06 | BDL | BDL | | 4.5 | 53310 | 4741 | 49 |
|  |  | 100 | 2.29 | 19.457 | 36.61 | 26.14 | 207.4 | 1.28 | 0.08 | BDL | | 3.5 | 50941 | 744 | 63 |
|  |  | 120 | 1.98 | 19.124 | 36.622 | 26.236 | 203 | 2.12 | 0.03 | BDL | | 2.2 | 22607 | 1293 | 202 |
|  |  | 140 | 2.63 | 18.765 | 36.613 | 26.323 | 204.3 | 2.72 | 0.01 | 0.06 | | 2 | 1008 | 144 | 88 |
|  |  | 160 | 1.69 | 18.469 | 36.585 | 26.377 | 209.9 | 2.81 | 0.01 | 0.07 | | 1.3 | 153 | 315 | 16 |
|  |  | 180 | 1.78 | n.d. | n.d. | n.d. | n.d. | n.d. | n.d. | n.d. | | n.d. | n.d. | n.d. | n.d. |
|  |  | 200 | 1.70 | 18.31 | 36.583 | 26.417 | 211.5 | 3.02 | 0.01 | 0.07 | | 1.4 | 16 | 293 | 7 |
|  |  | 300 | 2.37 | 17.887 | 36.536 | 26.491 | 200.8 | 4.71 | BDL | 0.18 | | 0.6 | n.d. | n.d. | n.d. |
|  |  | 400 | 2.46 | 16.648 | 36.314 | 26.624 | 193.7 | 6.11 | BDL | 0.26 | | 0.4 | n.d. | n.d. | n.d. |
|  |  | 500 | 1.76 | 15.992 | 36.271 | 26.747 | 222.3 | 7.5 | BDL | 0.34 | | n.d. | n.d. | n.d. | n.d. |
|  |  | 600 | 1.63 | 15.937 | 36.267 | 26.761 | 226.8 | 5.46 | n.d. | 0.26 | | n.d. | n.d. | n.d. | n.d. |
|  |  | 700 | 2.66 | 15.569 | 36.184 | 26.784 | 222.9 | 6.1 | n.d. | 0.3 | | n.d. | n.d. | n.d. | n.d. |
|  |  | 800 | 2.25 | 13.197 | 35.76 | 26.966 | 185.2 | 12.15 | n.d. | 0.72 | | 0.4 | n.d. | n.d. | n.d. |
|  |  | 900 | 1.73 | 10.005 | 35.308 | 27.211 | 149.6 | 21.57 | n.d. | 1.38 | | n.d. | n.d. | n.d. | n.d. |
|  |  | 1000 | 1.61 | 7.701 | 35.135 | 27.441 | 173.9 | 22.26 | n.d. | 1.45 | | n.d. | n.d. | n.d. | n.d. |
| 2010 | March | 0 | 2.53 | 19.598 | 36.709 | 26.174 | 223.5 | 0.27 | 0.03 | BDL | | 5.6 | 36011 | 17730 | 358 |
|  |  | 20 | 2.74 | 19.495 | 36.704 | 26.198 | 221.7 | 0.25 | 0.04 | BDL | | 5.4 | 34612 | 16869 | 370 |
|  |  | 40 | 2.57 | 19.428 | 36.693 | 26.208 | 222.7 | 0.37 | 0.04 | BDL | | 5.8 | 30844 | 17254 | 503 |
|  |  | 60 | 3.54 | 19.346 | 36.681 | 26.221 | 220.9 | 0.7 | 0.1 | BDL | | 5.5 | 17423 | 10697 | 279 |
|  |  | 80 | 2.49 | 19.207 | 36.669 | 26.249 | 219.7 | 0.88 | 0.13 | BDL | | 6.1 | 11842 | 7392 | 261 |
|  |  | 100 | 1.98 | 19.19 | 36.681 | 26.263 | 218.3 | 0.98 | 0.14 | BDL | | 5.2 | 8557 | 6401 | 249 |
|  |  | 120 | 2.56 | 19.028 | 36.663 | 26.293 | n.d. | 1.09 | 0.18 | BDL | | 5.4 | 4167 | 3432 | 193 |
|  |  | 140 | 2.83 | 18.988 | 36.657 | 26.299 | 219.2 | 1.06 | 0.2 | BDL | | 4.2 | 2853 | 2743 | 171 |
|  |  | 160 | 2.44 | 18.974 | 36.654 | 26.301 | 218.3 | 1.2 | 0.17 | BDL | | 3.8 | 2138 | 2350 | 174 |
|  |  | 200 | 2.06 | 18.753 | 36.618 | 26.333 | 208.2 | 2.24 | n.d. | BDL | | 2.3 | 616 | 974 | 165 |
|  |  | 250 | 2.23 | 18.42 | 36.575 | 26.386 | 215.8 | 2.05 | n.d. | BDL | | 2.1 | 377 | 722 | 227 |
|  |  | 300 | 2.57 | 18.171 | 36.55 | 26.431 | n.d. | n.d. | n.d. | n.d. | | n.d. | n.d. | n.d. | n.d. |
|  |  | 400 | 1.66 | 17.808 | 36.499 | 26.487 | n.d. | n.d. | n.d. | n.d. | | n.d. | n.d. | n.d. | n.d. |
| 2010 | September | 0 | 1.69 | 28.2 | 36.218 | 23.248 | 199.2 | 0.07 | BDL | BDL | | 5.8 | 37927 | 6233 | 65 |
|  |  | 20 | 1.76 | 27.646 | 36.208 | 23.423 | 200.5 | 0.07 | BDL | BDL | | 4.4 | 43280 | 7097 | 64 |
|  |  | 40 | 2.10 | 25.094 | 36.624 | 24.545 | 210.7 | 0.08 | BDL | BDL | | 4.1 | 77512 | 6855 | 79 |
|  |  | 60 | 1.42 | 22.559 | 36.72 | 25.372 | 213 | 0.06 | BDL | BDL | | 4 | 109831 | 5886 | 186 |
|  |  | 80 | 3.35 | 20.894 | 36.705 | 25.827 | 210.3 | 0.36 | 0.17 | BDL | | 3.1 | 85103 | 846 | 445 |
|  |  | 100 | 3.29 | 19.963 | 36.681 | 26.061 | 205.2 | 1.18 | 0.03 | BDL | | 2.7 | 25298 | 122 | 158 |
|  |  | 120 | 1.91 | 19.524 | 36.667 | 26.167 | 204.9 | 1.59 | 0.02 | 0.09 | | 2.5 | 3408 | 66 | 48 |
|  |  | 140 | 1.93 | 19.007 | 36.616 | 26.262 | 193.7 | 3.05 | 0.02 | 0.11 | | 2.1 | 72 | 13 | 8 |
|  |  | 160 | 1.85 | 18.783 | 36.621 | 26.325 | 192 | 3.6 | 0.01 | 0.1 | | 1.6 | 7 | 13 | 4 |
|  |  | 200 | 2.19 | 18.396 | 36.581 | 26.394 | 200.5 | 3.49 | 0.01 | 0.13 | | 1.6 | 7 | 7 | 2 |
|  |  | 250 | 1.73 | 18.19 | 36.571 | 26.441 | 204.6 | 3.76 | 0.01 | 0.17 | | 1.5 | 32 | 6 | 0 |
|  |  | 300 | 2.29 | 17.977 | 36.543 | 26.474 | 205.6 | 4.35 | 0.01 | 0.16 | | 0.9 | n.d. | n.d. | n.d. |
|  |  | 400 | 2.11 | 17.459 | 36.47 | 26.55 | 209.1 | 4.96 | 0.01 | 0.17 | | 0.9 | n.d. | n.d. | n.d. |
| 2011 | March | 0 | 2.47 | 19.483 | 36.656 | 26.164 | 232 | 0.14 | 0.03 | BDL | | 8.1 | 2012 | 11033 | 278 |
|  |  | 20 | 2.34 | 19.459 | 36.714 | 26.215 | 232.4 | 0.12 | 0.03 | BDL | | 7.7 | 2126 | 11532 | 341 |
|  |  | 40 | 1.73 | 18.921 | 36.677 | 26.327 | 231.9 | 0.13 | 0.03 | BDL | | 7.3 | 2087 | 12767 | 445 |
|  |  | 60 | 1.78 | 18.898 | 36.676 | 26.333 | 229 | 0.39 | 0.09 | BDL | | 7 | 1568 | 9212 | 364 |
|  |  | 80 | 1.73 | 18.862 | 36.677 | 26.344 | 224.8 | 0.87 | 0.2 | BDL | | 7.4 | 2460 | 12315 | 199 |
|  |  | 100 | 1.63 | 18.712 | 36.67 | 26.378 | 224 | 1.06 | 0.2 | BDL | | 6.8 | 2612 | 15571 | 212 |
|  |  | 120 | 1.53 | 18.677 | 36.667 | 26.385 | 224 | 1.39 | 0.18 | 0.05 | | 4.8 | 1809 | 9280 | 121 |
|  |  | 140 | 1.54 | 18.647 | 36.663 | 26.391 | 218.3 | 1.85 | 0.1 | 0.07 | | 4.5 | 906 | 3072 | 40 |
|  |  | 160 | 1.17 | 18.567 | 36.658 | 26.408 | 216.6 | 2.12 | 0.08 | 0.08 | | 4.1 | 575 | 1611 | 22 |
|  |  | 180 | 1.47 | 18.547 | 36.655 | 26.412 | n.d. | n.d. | n.d. | n.d. | | n.d. | n.d. | n.d. | n.d. |
|  |  | 200 | 1.39 | 18.525 | 36.652 | 26.416 | 216.7 | 2.28 | 0.05 | 0.1 | | 4.2 | 360 | 1029 | 18 |
|  |  | 250 | 1.45 | 18.296 | 36.617 | 26.449 | 212.4 | 3 | 0.03 | 0.1 | | 3.1 | 283 | 602 | 12 |
|  |  | 300 | 1.57 | 18.215 | 36.614 | 26.469 | 218.9 | 2.47 | 0.04 | 0.09 | | 2.5 | n.d. | n.d. | n.d. |
|  |  | 400 | 1.68 | 18.081 | 36.593 | 26.491 | 217.2 | 2.88 | 0.02 | 0.13 | | 2.4 | n.d. | n.d. | n.d. |
|  |  | 500 | 1.96 | 17.075 | 36.391 | 26.586 | 200.8 | 5.12 | 0.01 | 0.21 | | n.d. | n.d. | n.d. | n.d. |
|  |  | 600 | 2.10 | 16.214 | 36.225 | 26.665 | 186.5 | 8.97 | n.d. | n.d. | | n.d. | n.d. | n.d. | n.d. |
|  |  | 700 | 2.35 | 14.677 | 35.949 | 26.799 | 167.5 | 14.05 | n.d. | n.d. | | n.d. | n.d. | n.d. | n.d. |
|  |  | 800 | 1.83 | 12.157 | 35.561 | 27.017 | 152.5 | 19.16 | n.d. | n.d. | | 1.1 | n.d. | n.d. | n.d. |
|  |  | 900 | 2.51 | 9.893 | 35.291 | 27.217 | 154.7 | 22.82 | n.d. | n.d. | | n.d. | n.d. | n.d. | n.d. |
|  |  | 1000 | 1.11 | 7.631 | 35.134 | 27.45 | 178.9 | 22.46 | n.d. | n.d. | | n.d. | n.d. | n.d. | n.d. |
| 2011 | September | 0 | 1.51 | 27.694 | 36.56 | 23.671 | 201.8 | BDL | BDL | BDL | | 5.4 | 20620 | 12079 | 57 |
|  |  | 20 | 1.31 | 27.803 | 36.693 | 23.737 | 199.8 | BDL | BDL | BDL | | 5.5 | 26776 | 9964 | 55 |
|  |  | 40 | 2.00 | 26.099 | 36.602 | 24.216 | 232 | BDL | BDL | BDL | | 5.7 | 73087 | 6274 | 80 |
|  |  | 60 | 4.00 | 22.832 | 36.768 | 25.329 | 231.7 | BDL | 0.01 | BDL | | 5.9 | 92280 | 7023 | 100 |
|  |  | 80 | 3.22 | 21.493 | 36.758 | 25.702 | 224.3 | BDL | BDL | BDL | | 6.1 | 118515 | 6479 | 179 |
|  |  | 100 | 3.05 | 20.394 | 36.716 | 25.972 | 211.8 | BDL | BDL | BDL | | 5.1 | 94467 | 4135 | 269 |
|  |  | 120 | 1.97 | 19.514 | 36.673 | 26.174 | 200.4 | 0.49 | 0.14 | BDL | | 4.7 | 54141 | 449 | 356 |
|  |  | 140 | 2.75 | 18.979 | 36.65 | 26.296 | 207.1 | 0.64 | 0.16 | 0.05 | | 3.2 | 28364 | 205 | 186 |
|  |  | 160 | 2.32 | 18.672 | 36.63 | 26.36 | 204.3 | 2.34 | 0.03 | 0.07 | | 2.3 | 10145 | 184 | 107 |
|  |  | 180 | 1.94 | 18.528 | 36.619 | 26.389 | n.d. | n.d. | n.d. | n.d. | | n.d. | n.d. | n.d. | n.d. |
|  |  | 200 | 1.21 | 18.413 | 36.612 | 26.414 | 205 | 3.17 | 0.01 | 0.14 | | 2.1 | 1174 | 222 | 32 |
|  |  | 250 | 2.29 | 18.189 | 36.59 | 26.456 | 206.4 | 2.99 | 0.01 | 0.14 | | n.d. | 28 | 202 | 31 |
|  |  | 300 | 2.37 | 18.073 | 36.578 | 26.477 | 207 | 3.66 | 0.02 | 0.08 | | 1.6 | n.d. | n.d. | n.d. |
|  |  | 400 | 1.85 | 17.744 | 36.523 | 26.52 | 201.9 | 4.78 | 0.02 | 0.2 | | 0.9 | n.d. | n.d. | n.d. |
|  |  | 500 | 1.69 | 17.034 | 36.389 | 26.594 | 189.7 | 7.5 | 0.01 | 0.27 | | n.d. | n.d. | n.d. | n.d. |
|  |  | 600 | 2.45 | 15.444 | 36.084 | 26.731 | 175.4 | 11.7 | n.d. | 0.49 | | n.d. | n.d. | n.d. | n.d. |
|  |  | 700 | 0.81 | 13.659 | 35.789 | 26.891 | 165.3 | 15.87 | n.d. | 0.7 | | n.d. | n.d. | n.d. | n.d. |
|  |  | 800 | 3.07 | 11.137 | 35.438 | 27.111 | 148.9 | 21.6 | n.d. | 0.99 | | 0.9 | n.d. | n.d. | n.d. |
|  |  | 900 | 6.49 | 9.082 | 35.226 | 27.299 | 158.9 | 23.18 | n.d. | 1.19 | | n.d. | n.d. | n.d. | n.d. |
|  |  | 1000 | 1.86 | 6.868 | 35.107 | 27.535 | 188.7 | 21.9 | n.d. | 1.28 | | n.d. | n.d. | n.d. | n.d. |
