# Supplementary material for: Deep sequencing of the viral phoH gene reveals temporal variation, depth-specific composition, and persistent dominance of the same viral phoH genes in the Sargasso Sea
Source: PeerJ. 2015 Jun 16;3:e997. doi: 10.7717/peerj.997 (PMC4476143; doi:10.7717/peerj.997)
Supplement: Supplemental Information 1 [file peerj-03-997-s012.docx]

Rank-abundance plots constructed for each of the 85 samples revealed distributions that appeared to come from the same family of functions, yet were visibly distinct. Each rank-abundance distribution was compared to power law, exponential, and lognormal distributions to determine the best fit to the data. All three functional forms fit the data reasonably well but not well enough to claim true representation of the empirical distributions (see plot of sum squared errors, Supplemental Fig. S4). Instead, these comparisons revealed a strong correlation (R^2^ = 0.973; Supplemental Fig. S5) between the rate parameter in the exponential fit (k from abundance = C*exp(-k*rank)) and the power parameter in the power law fit (b from abundance = D*rank^(-b)). This correlation is remarkable considering the fact that exponential and power law distributions are very different functional forms, but can be explained by the extreme dominance of just a few OTUs in each sample, followed by a steep decline in OTU abundance.
